# Whole-exome sequencing of *BRCA-*negative breast cancer patients and case–control analyses identify variants associated with breast cancer susceptibility

**DOI:** 10.1186/s40246-022-00435-7

**Published:** 2022-11-23

**Authors:** Ning Yuan Lee, Melissa Hum, Aseervatham Anusha Amali, Wei Kiat Lim, Matthew Wong, Matthew Khine Myint, Ru Jin Tay, Pei-Yi Ong, Jens Samol, Chia Wei Lim, Peter Ang, Min-Han Tan, Soo-Chin Lee, Ann S. G. Lee

**Affiliations:** 1 Division of Cellular and Molecular Research, Humphrey Oei Institute of Cancer Research, National Cancer Centre Singapore, 11 Hospital Crescent, Singapore, 169610 Singapore; 2 Lucence Diagnostics Pte Ltd, 211 Henderson Road, Singapore, 159552 Singapore; 3 Department of Hematology-Oncology, National University Cancer Institute, Singapore (NCIS), National University Health System, 5 Lower Kent Ridge Road, Singapore, 119074 Singapore; 4 Medical Oncology Department, Tan Tock Seng Hospital, 11 Jalan Tan Tock Seng, Singapore, 308433 Singapore; 5 Johns Hopkins University, Baltimore, MD 21218 USA; 6 Department of Personalised Medicine, Tan Tock Seng Hospital, 11 Jalan Tan Tock Seng, Singapore, 308433 Singapore; 7 Oncocare Cancer Centre, Gleneagles Medical Centre, 6 Napier Road, Singapore, 258499 Singapore; 8 Department of Medicine, Yong Loo Lin School of Medicine, National University of Singapore, 10 Medical Dr, Singapore, 117597 Singapore; 9 Cancer Science Institute, Singapore (CSI), National University of Singapore, 14 Medical Dr, Singapore, 117599 Singapore; 10 Department of Physiology, Yong Loo Lin School of Medicine, National University of Singapore, 2 Medical Drive, Singapore, 117593 Singapore; 11 SingHealth Duke-NUS Oncology Academic Clinical Programme (ONCO ACP), Duke-NUS Graduate Medical School, 8 College Road, Singapore, 169857 Singapore

**Keywords:** Breast cancer, *BRCA*1/2 negative, Whole-exome sequencing, Germline variants, Case–control analysis

## Abstract

**Background:**

For the majority of individuals with early-onset or familial breast cancer referred for genetic testing, the genetic basis of their familial breast cancer remains unexplained. To identify novel germline variants associated with breast cancer predisposition, whole-exome sequencing (WES) was performed.

**Methods:**

WES on 290 *BRCA1/BRCA2*-negative Singaporeans with early-onset breast cancer and/or a family history of breast cancer was done. Case–control analysis against the East-Asian subpopulation (EAS) from the Genome Aggregation Database (gnomAD) identified variants enriched in cases, which were further selected by occurrence in cancer gene databases. Variants were further evaluated in repeated case–control analyses using a second case cohort from the database of Genotypes and Phenotypes (dbGaP) comprising 466 early-onset breast cancer patients from the United States, and a Singapore SG10K_Health control cohort.

**Results:**

Forty-nine breast cancer-associated germline pathogenic variants in 37 genes were identified in Singapore cases versus gnomAD (EAS). Compared against SG10K_Health controls, 13 of 49 variants remain significantly enriched (False Discovery Rate (FDR)-adjusted *p* < 0.05). Comparing these 49 variants in dbGaP cases against gnomAD (EAS) and SG10K_Health controls revealed 23 concordant variants that were significantly enriched (FDR-adjusted *p* < 0.05). Fourteen variants were consistently enriched in breast cancer cases across all comparisons (FDR-adjusted *p* < 0.05). Seven variants in *GPRIN2, NRG1, MYO5A, CLIP1*, *CUX1, GNAS* and *MGA* were confirmed by Sanger sequencing.

**Conclusions:**

In conclusion, we have identified pathogenic variants in genes associated with breast cancer predisposition. Importantly, many of these variants were significant in a second case cohort from dbGaP, suggesting that the strategy of using case–control analysis to select variants could potentially be utilized for identifying variants associated with cancer susceptibility.

**Supplementary Information:**

The online version contains supplementary material available at 10.1186/s40246-022-00435-7.

## Introduction

Breast cancer (BC) is the most common malignancy and the leading cause of cancer-associated mortality among women worldwide [1]. It accounts for one in four cancer cases among women and one in six cancer deaths, ranking first in the vast majority of countries for incidence [1]. Approximately, 10–20% of all BC patients have a family history of cancer with multiple family members affected across generations [2]. Germline mutations in specific genes such as *BRCA1*, *BRCA2, CDH1, PALB2, PTEN* and *TP53* confer an increased risk of developing BC [3].

Recent advances in next-generation sequencing have led to reduced costs for multigene panel testing of cancer predisposition genes for individuals referred for genetic testing, resulting in a higher uptake of testing. However, it is estimated that pathogenic variants in known cancer predisposition genes only account for around 25% of hereditary BC cases [4, 5].

Whole-exome sequencing (WES) is revolutionizing our ability to identify novel genetic variants associated with cancer predisposition. To date, multiple candidate BC predisposition genes have been identified by WES, predominantly from studies on women of European ancestry [6, 7].

Here, we aimed to identify novel candidate BC predisposition genes and variants by performing WES on germline DNA from Asian BC patients referred for cancer genetic risk assessment but who were *BRCA1/2*-negative. Pathogenic variants identified from WES were filtered and prioritized using in silico bioinformatic tools, followed by case–control analysis and only significant variants in known cancer genes were selected for further analysis. Notably, we have identified pathogenic variants in our cases that had a statistically significant difference in frequency as compared to the Genome Aggregation Database (gnomAD) East-Asian (EAS) controls and Singaporean controls [8].

## Results

### Demographics and clinical information on the study population

Information on the demographics, age at diagnosis, ethnicity, family history, and clinicopathological characteristics of the 290 BC cases are provided in Table 1. The study population consisted of only females, and a large proportion were Chinese (69.3%). The age of first cancer diagnosis ranged from 19 to 75 years, with a mean and median age of 37.5 and 37 years, respectively. Of 290 patients, 65 (22.4%) presented with a family history (including first-degree, second-degree, and third-degree relatives) of BC, 23 (7.9%) with a family history of other cancers and 218 (75.2%) with no family history of breast or any other cancers (Table 1, Additional file 1: Fig. S1). Of the 290 BC cases, 225 patients (77.6%) had early-onset breast cancer (≤ 40 years).

**Table 1:** Demographics, clinical characteristics, and family history of patients

Patient characteristics	Number (%)
Total number of patients	290 (100.0)
*Race/ethnicity*	
Chinese	201 (69.3)
Malay	19 (6.6)
Indian	17 (5.9)
Caucasian	4 (1.4)
Filipino	4 (1.4)
Indonesian	3 (1.0)
Vietnamese	3 (1.0)
Arab	2 (0.7)
Bengali	1 (0.3)
Burmese	1 (0.3)
Gujarati	1 (0.3)
Tamil	1 (0.3)
Others	30 (10.3)
Not reported	3 (1.0)
*Age at breast cancer diagnosis*	
Median	37.0 yrs
Range	19–75 yrs
≤ 40	225 (77.6)
≥ 41	65 (22.4)
*Family history of breast cancer (n = 138)*	
At least first-degree	78 (26.9)
At least second-degree	43 (14.8)
At least third-degree	16 (5.5)
Unspecified	1 (0.3)
*Family history of other cancers (n = 48)*	
At least first-degree	20 (6.9)
At least second-degree	28 (9.7)
*Histology*	
Ductal carcinoma in situ (DCIS)	14 (4.8)
Infiltrating ductal carcinoma (IDC)	180 (62.1)
Infiltrating lobular carcinoma (ILC)	7 (2.4)
Mucinous carcinoma	9 (3.1)
Medullary carcinoma	2 (0.7)
Encapsulated papillary carcinoma	1 (0.3)
Tubulolobular carcinoma	1 (0.3)
DCIS + Lobular carcinoma in situ	1 (0.3)
DCIS + IDC + Mucinous	2 (0.7)
IDC + ILC	5 (1.7)
IDC + Invasive micropapillary carcinoma	1 (0.3)
IDC + Medullary	1 (0.3)
Subtype not defined^a^	66 (22.8)
*Hormone and HER2 status*	
ER	
Positive	182 (62.8)
Negative	68 (23.4)
Not tested/unknown^a^	40 (13.8)
PR	
Positive	158 (54.5)
Negative	90 (31)
Not tested/unknown^a^	42 (14.5)
HER2	
Positive	77 (26.6)
Negative	150 (51.7)
Not tested/unknown/equivocal^a^	63 (21.7)

^a^Clinical information for some patients was unavailable from one of the sites of this study due to the Institutional Review Board (IRB) approval obtained

### Filtering of candidate variants

**Fig. 1:**
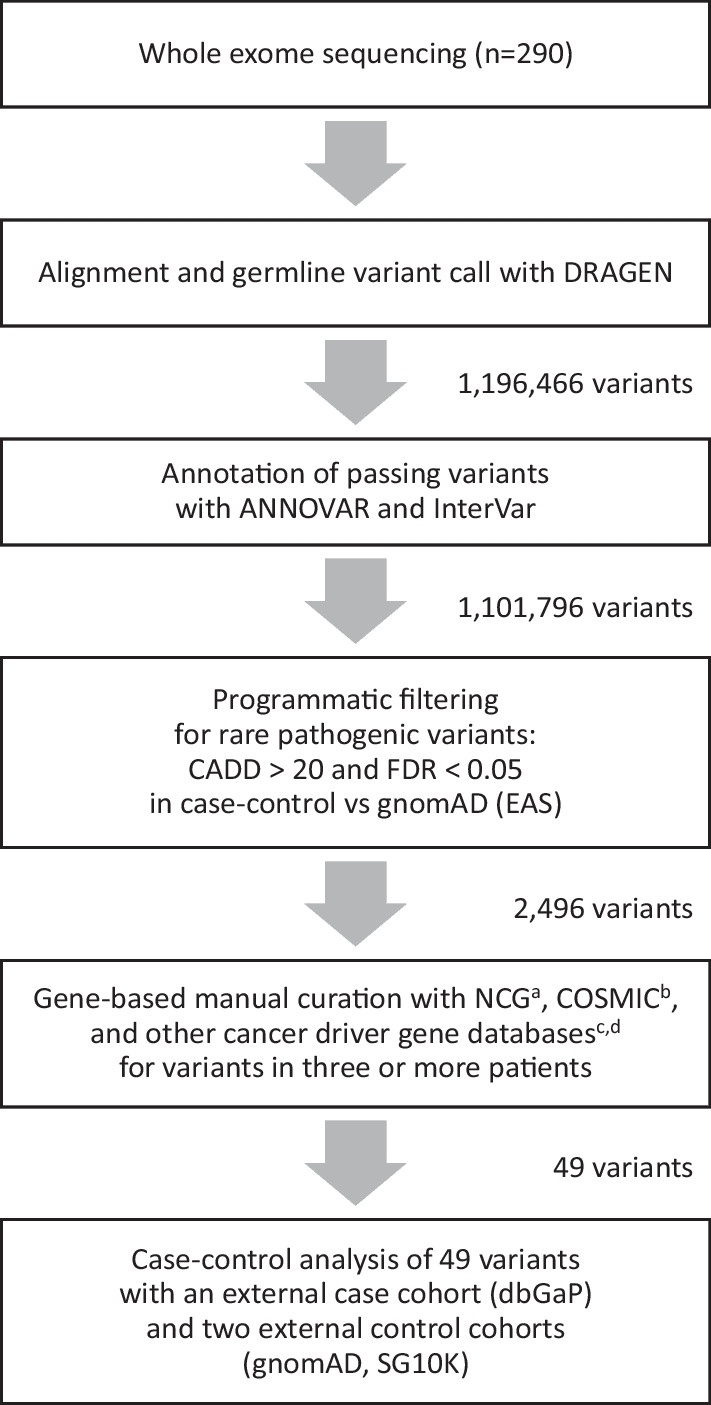
Study design for the selection of variants and genes. ^a^List of known or candidate cancer genes in the Network of Cancer Genes [9]. ^b^The Cancer Gene Census list of the Catalogue of Somatic Mutations in Cancer (COSMIC) [10]. ^c^List of cancer driver genes from Bailey et al. [38]. ^d^List of cancer driver genes inferred with nucleotide context from Dietlein et al. [11]

Whole exome sequencing of 290 BC patients revealed 1,196,466 variants before filtering. Among these, 1,101,796 (92.1%) passed Dynamic Read Analysis for GENomics (DRAGEN) quality-control checks. Further filtering to retain functional variants with gnomAD (EAS) minor allele frequency (MAF) less than 1%, predicted pathogenic variants with scaled Combined Annotation-Dependent Depletion (CADD) score greater than 20, and variants in the known or predicted cancer gene lists in the Network of Cancer Genes (NCG) database, left only 2,496 variants (0.2% of the total; Fig. 1).

The genes of our shortlisted variants were further prioritized using cancer genes databases such as Catalogue of Somatic Mutations in Cancer (COSMIC), cancer driver genes based on nucleotide context, and computationally discovered and experimentally validated cancer driver genes [9] (Additional file 4: Table S1). Finally, we shortlist only variants that were present in three or more patients. All variants were checked with IGV (Additional files 1, 2: Figs. S1, S2).

### Identification of pathogenic germline variants

**Fig. 2:**
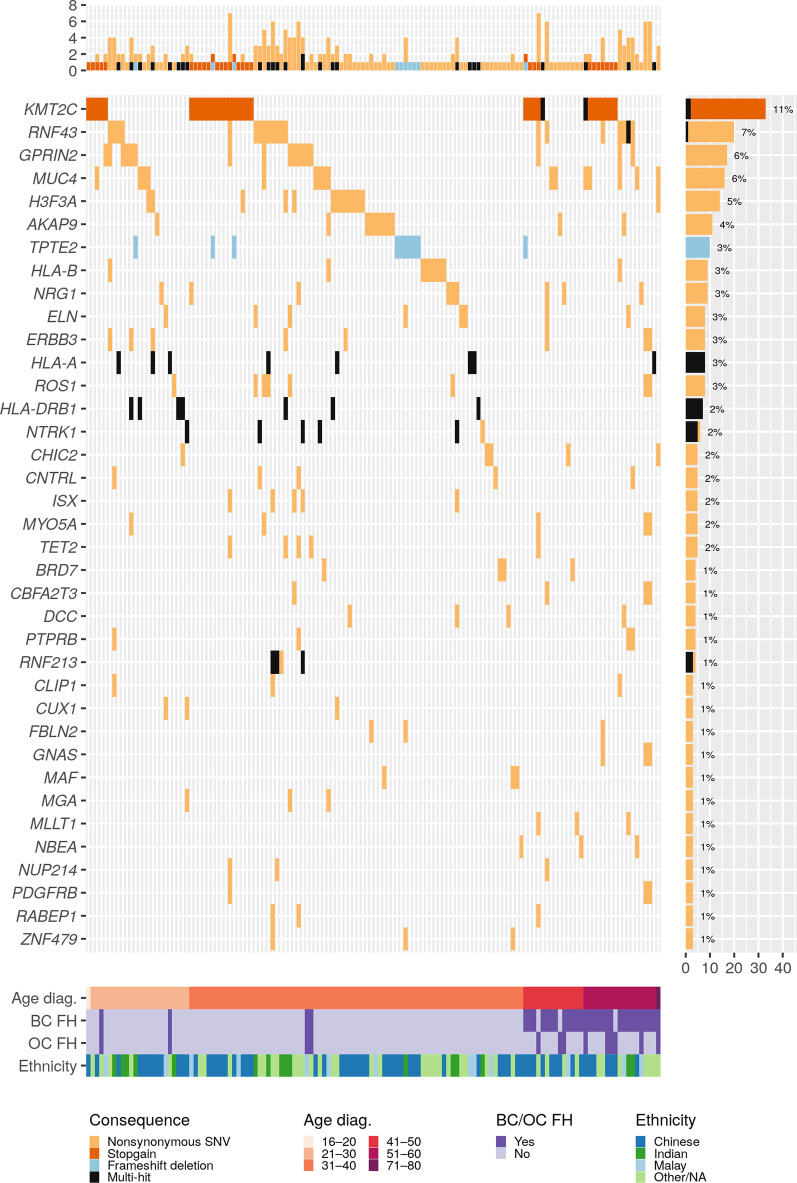
Oncoplot of variants in prioritized candidate genes, showing the type and frequency of each variant. Rows represent genes and each column represents one case. Rows (bottom) show the age at diagnosis (diag), family history (FH) status for breast cancer (BC) and ovarian cancer (OC) and ethnicity for each case

In total, we discovered 49 prioritized variants in 37 prioritized genes across 134 patients (Fig. 2; Additional file 4: Table S2). Most of these variants are nonsynonymous single nucleotide variants (SNVs) (42 variants, or 85.7%), with one frameshift insertion (2.0%), three frameshift deletions (6.1%), and three stop-gains (6.1%). Frameshift insertions, deletions, and stop-gains were prioritized regardless of their CADD score.

All 42 nonsynonymous SNVs had CADD scores greater than 20. The remaining 7 variants which were not nonsynonymous SNVs also had CADD scores greater than 20, except for a frameshift deletion variant in *HLA-A*. Thirty variants were classified as variants of uncertain significance (VUS) (61.2%), two stop-gain mutations in *KMT2C* were considered pathogenic (4.1%), and the remaining variants were benign (14 variants, or 28.6%) or likely benign (3 variants, or 6.1%) (Table 2).

**Table 2:** Predicted pathogenicity and classifications from databases for 49 selected variants in 37 genes

Gene	HGVS	RefSNP	Consequence	Protein Change	CADD Score	ClinVar	ACMG Classification (InterVar)
*KMT2C*	NM_170606.3:c.C2689T	rs772146328	Stopgain	p.R897X	39.0	–	Pathogenic
*KMT2C*	NM_170606.3:c.C2710T	rs200662726	Stopgain	p.R904X	37.0	–	Pathogenic
*RNF43*	NM_001305545.1:c.C311T	rs2680701	Nonsyn. SNV	p.P104L	24.0	–	Benign
*RNF43*	NM_001305545.1:c.G1589C	rs142097313	Nonsyn. SNV	p.R530P	26.5	–	Uncertain significance
*RNF43*	NM_001305545.1:c.G647A	rs34523089	Nonsyn. SNV	p.R216H	25.4	–	Benign
*GPRIN2*	NM_014696.4:c.C983G	rs4445576	Nonsyn. SNV	p.S328C	23.1	–	Benign
*MUC4*	NM_018406.7:c.G8461A	rs868560707	Nonsyn. SNV	p.D2821N	22.1	–	Uncertain significance
*H3F3A*	NM_002107.6:c.C344G	rs749423281	Nonsyn. SNV	p.A115G	28.8	–	Uncertain significance
*AKAP9*	NM_005751.4:c.T3430C	rs141039834	Nonsyn. SNV	p.C1144R	20.5	Conflicting interpretations of pathogenicity	Uncertain significance
*TPTE2*	NM_199254.2:c.483delT	–	Frameshift del	p.F161Lfs*15	22.0	–	Uncertain significance
*HLA-B*	NM_005514.8:c.A161G	rs9266183	Nonsyn. SNV	p.D54G	23.6	–	Uncertain significance
*NRG1*	NM_013962.2:c.G172A	rs113317778	Nonsyn. SNV	p.G58R	23.6	–	Benign
*ELN*	NM_001278913.2:c.G1498C	rs17855988	Nonsyn. SNV	p.G500R	23.2	Benign	Benign
*ERBB3*	NM_001982.3:c.A3355T	rs773123	Nonsyn. SNV	p.S1119C	23.8	–	Benign
*HLA-A*	NM_001242758.1:c.268delA	rs756231831	Frameshift del	p.N90Mfs*2	12.03	–	Uncertain significance
*HLA-A*	NM_001242758.1:c.C791T	rs41548917	Nonsyn. SNV	p.T264I	25.6	–	Uncertain significance
*HLA-A*	NM_001242758.1:c.G1055T	rs369261720	Nonsyn. SNV	p.S352I	23.6	–	Uncertain significance
*HLA-A*	NM_001242758.1:c.G565A	rs41562120	Nonsyn. SNV	p.V189M	22.1	–	Uncertain significance
*HLA-A*	NM_001242758.1:c.G684A	rs372503438	Stopgain	p.W228X	37.0	–	Uncertain significance
*HLA-A*	NM_001242758.1:c.T547C	rs758168864	Nonsyn. SNV	p.Y183H	24.4	–	Uncertain significance
*ROS1*	NM_002944.2:c.C3326T	rs2229079	Nonsyn. SNV	p.S1109L	21.1	–	Benign
*HLA-DRB1*	NM_002124.3:c.118_122del	rs756741350	Frameshift del	p.P40Efs*21	24.7	–	Uncertain significance
*HLA-DRB1*	NM_002124.3:c.126_127insTTAAGTTT	rs769556955	Frameshift ins	p.E43Lfs*40	24.6	–	Uncertain significance
*HLA-DRB1*	NM_002124.3:c.C301T	rs17885222	Nonsyn. SNV	p.R101W	25.5	–	Likely benign
*NTRK1*	NM_001012331.1:c.C1792T	rs6336	Nonsyn. SNV	p.H598Y	27.8	–	Benign
*NTRK1*	NM_001012331.1:c.G1820T	rs6339	Nonsyn. SNV	p.G607V	22.0	–	Benign
*CHIC2*	NM_012110.4:c.G36T	rs368360781	Nonsyn. SNV	p.E12D	22.2	–	Uncertain significance
*CNTRL*	NM_001330762.2:c.G1009A	rs17292952	Nonsyn. SNV	p.A337T	21.9	–	Uncertain significance
*ISX*	NM_001303508.2:c.G248A	rs8140287	Nonsyn. SNV	p.R83Q	34.0	–	Uncertain significance
*MYO5A*	NM_000259.3:c.A3960T	rs61731219	Nonsyn. SNV	p.R1320S	21.7	Benign	Benign
*TET2*	NM_001127208.2:c.C1088T	rs17253672	Nonsyn. SNV	p.P363L	23.3	–	Uncertain significance
*BRD7*	NM_001173984.3:c.A44C	–	Nonsyn. SNV	p.Y15S	22.8	–	Uncertain significance
*CBFA2T3*	NM_005187.6:c.G308C	rs61734177	Nonsyn. SNV	p.R103P	22.5	–	Benign
*DCC*	NM_005215.4:c.A3578G	rs375401214	Nonsyn. SNV	p.Q1193R	23.1	–	Uncertain significance
*PTPRB*	NM_001206971.3:c.C3412T	rs61754227	Nonsyn. SNV	p.R1138W	25.1	–	Uncertain significance
*RNF213*	NM_001256071.3:c.C12847A	rs62077764	Nonsyn. SNV	p.L4283I	23.1	–	Benign
*RNF213*	NM_001256071.3:c.C13945G	rs61745599	Nonsyn. SNV	p.L4649V	24.4	–	Benign
*CLIP1*	NM_001247997.1:c.C80T	rs34292795	Nonsyn. SNV	p.T27M	23.3	–	Likely benign
*CUX1*	NM_001202543.2:c.C3317T	rs782176246	Nonsyn. SNV	p.P1106L	24.8	–	Uncertain significance
*FBLN2*	NM_001998.3:c.G2569T	rs556004379	Nonsyn. SNV	p.V857L	29.0	–	Uncertain significance
*GNAS*	NM_016592.4:c.A266G	rs563844600	Nonsyn. SNV	p.E89G	23.8	–	Uncertain significance
*MAF*	NM_001031804.3:c.G655T	rs1030258012	Nonsyn. SNV	p.G219C	22.2	–	Uncertain significance
*MGA*	NM_001080541.2:c.C1883A	rs61736074	Nonsyn. SNV	p.P628Q	25.8	–	Uncertain significance
*MLLT1*	NM_005934.4:c.G889A	rs11880101	Nonsyn. SNV	p.A297T	24.8	–	Uncertain significance
*NBEA*	NM_015678.4:c.C2317A	–	Nonsyn. SNV	p.L773M	27.3	–	Uncertain significance
*NUP214*	NM_001318324.2:c.A2263G	rs61756081	Nonsyn. SNV	p.I755V	23.6	Benign	Uncertain significance
*PDGFRB*	NM_001355016.2:c.G1261A	rs41287110	Nonsyn. SNV	p.E421K	21.2	Benign	Benign
*RABEP1*	NM_001291581.2:c.G1755C	rs61735455	Nonsyn. SNV	p.M585I	22.6	–	Uncertain significance
*ZNF479*	NM_001370129.1:c.T1421C	rs200382632	Nonsyn. SNV	p.F474S	23.3	–	Likely benign

A dash (“–”) indicates that a variant does not have a RefSNP accession number

### Case–control analysis of the Singapore cases

Case–control analysis was performed for 49 selected variants for our Singaporean cases against the gnomAD (EAS) and SG10K_Health control cohorts (Table 3). Apart from the two variants in *BRD7* and *NBEA* that were not reported in gnomAD (EAS), all of our remaining 47 variants were significantly enriched in our cohort as compared to gnomAD (EAS). In the SG10K_Health control cohort, seven of our 49 selected variants were absent, including the aforementioned variants in *BRD7* and *NBEA;* and additional variants in *KMT2C, GPRIN2, H3F3A,* and *MAF.* Of the remaining 42 variants which could be found in SG10K_Health, 13 were significantly enriched at α = 0.05 in our cohort versus SG10K_Health (Table 3).

**Table 3:** Allele frequencies and case–control association analysis of 49 variants in 37 selected candidate genes

Gene	HGVS	Number of Patients	Allele Frequency (%)				Odds Ratio (95% CI)			
			Cases		Controls		Our Study vs		dbGaP phs000822.v1.p1 vs	
			Our Study	dbGaP phs000822.v1.p1	gnomAD (EAS)	SG10K_Health	gnomAD (EAS)	SG10K_Health	gnomAD (EAS)	SG10K_Health
*KMT2C*	NM_170606.3:c.C2689T	7	1.21	0.11	0.02	–	72.88 (16.54–434.73***) *p* = 1.9e−08	–	6.40 (0.12–79.79) *p* = 0.21	–
*KMT2C*	NM_170606.3:c.C2710T	28	4.83	2.37	0.20	–	25.25 (14.43–44.09***) *p* = 1.6e−23	–	12.07 (6.60–21.73***) *p* = 4.6e−14	–
*RNF43*	NM_001305545.1:c.C311T	7	1.21	17.81	0.06	1.64	19.99 (6.54–56.74***) *p* = 2.7e−06	0.73 (0.29–1.54) *p* = 0.58	351.43 (191.23–741.46***) *p* = 4.5e−206	12.99 (10.55–15.96***) *p* = 7e−100
*RNF43*	NM_001305545.1:c.G1589C	3	0.52	–	0.00	1.01	Inf (14.25–Inf***) *p* = 5.3e−05	0.51 (0.10–1.52) *p* = 0.48	–	–
*RNF43*	NM_001305545.1:c.G647A	11	1.90	8.76	0.03	1.33	71.69 (21.14–308.16***) *p* = 2.3e−12	1.43 (0.70–2.63) *p* = 0.36	355.70 (132.69–1317.96***) *p* = 3.3e−93	7.11 (5.40–9.29***) *p* = 1.1e−34
***GPRIN2***	**NM_014696.4:c.C983G**	**17**	**2.93**	**20.92**	**0.10**	**–**	30.03 (14.69–60.72***) *p* = 5.4e−16	**–**	263.13 (165.05–446.60***) *p* = 1e−243	**–**
*MUC4*	NM_018406.7:c.G8461A	16	3.60	–	0.00	0.04	Inf (112.61–Inf***) *p* = 1.7e−26	104.51 (37.90–359.64***) *p* = 3e−23	–	–
*H3F3A*	NM_002107.6:c.C344G	14	2.41	0.65	0.71	–	3.47 (1.82–6.11***) *p* = 0.00027	–	0.91 (0.33–2.06) *p* = 1	–
*AKAP9*	NM_005751.4:c.T3430C	11	1.90	–	0.43	1.62	4.52 (2.16–8.56***) *p* = 0.00016	1.18 (0.58–2.15) *p* = 0.68	–	–
***TPTE2***	**NM_199254.2:c.483delT**	**10**	**1.77**	**–**	**0.08**	**0.04**	23.88 (9.05–62.22***) *p* = 7.5e−09	48.20 (15.81–161.69***) *p* = 1.1e−10	**–**	**–**
*HLA-B*	NM_005514.8:c.A161G	9	1.55	5.71	0.30	1.05	5.32 (2.30–10.92***) *p* = 0.00023	1.49 (0.62–3.24) *p* = 0.37	20.43 (13.65–30.55***) *p* = 1e−40	5.71 (3.56–9.33***) *p* = 2.9e−14
***NRG1***	**NM_013962.2:c.G172A**	**9**	**1.55**	**33.33**	**0.06**	**3.40**	25.36 (3.50–1107.34***) *p* = 9.4e−05	0.45 (0.20–0.86*) *p* = 0.02	705.43 (31.16–4.50e15***) *p* = 4.6e−05	14.20 (1.28–99.40*) *p* = 0.02
*ELN*	NM_001278913.2:c.G1498C	8	1.38	8.91	0.03	1.25	55.70 (16.02–218.37***) *p* = 3.1e−09	1.10 (0.47–2.23) *p* = 0.76	387.67 (159.04–1222.15***) *p* = 7.6e−106	7.72 (5.88–10.05***) *p* = 2.1e−38
*ERBB3*	NM_001982.3:c.A3355T	8	1.38	11.51	0.11	1.60	13.27 (5.06–31.33***) *p* = 3.4e−06	0.86 (0.37–1.73) *p* = 0.9	123.49 (76.10–207.51***) *p* = 3.4e−123	7.98 (6.26–10.10***) *p* = 1.3e−49
*HLA-A*	NM_001242758.1:c.268delA	8	1.38	0.21	0.04	0.94	34.34 (11.19–105.30***) *p* = 2.4e–08	1.47 (0.61–3.07) *p* = 0.36	5.28 (0.55–26.52) *p* = 0.087	0.23 (0.03–0.85*) *p* = 0.028
*HLA-A*	NM_001242758.1:c.C791T	8	1.38	–	0.13	1.95	10.66 (4.15–24.39***) *p* = 1.2e−05	0.70 (0.30–1.42) *p* = 0.53	–	–
*HLA-A*	NM_001242758.1:c.G1055T	8	1.38	0.11	0.14	3.46	10.30 (4.03–23.44***) *p* = 1.5e−05	0.39 (0.17–0.79**) *p* = 0.0075	0.79 (0.02–4.81) *p* = 1	0.03 (0.00–0.17***) *p* = 3.5e−12
*HLA-A*	NM_001242758.1:c.G565A	8	1.38	0.11	0.15	7.04	9.50 (3.74–21.43***) *p* = 2.2e−05	0.18 (0.08–0.37***) *p* = 1.9e−08	0.73 (0.02–4.41) *p* = 1	0.01 (0.00–0.08***) *p* = 3.1e−24
*HLA-A*	NM_001242758.1:c.G684A	8	1.38	–	0.06	1.31	25.19 (8.76–69.03***) *p* = 1.2e−07	1.06 (0.45–2.15) *p* = 0.89	–	–
*HLA-A*	NM_001242758.1:c.T547C	3	0.52	0.11	0.02	0.21	34.04 (4.55–254.09***) *p* = 0.00076	2.51 (0.39–12.93) *p* = 0.26	7.03 (0.13–87.78) *p* = 0.2	0.52 (0.01–4.64) *p* = 1
*ROS1*	NM_002944.2:c.C3326T	8	1.55	7.83	0.27	1.64	5.81 (2.51–11.93***) *p* = 0.00013	0.94 (0.43–1.83) *p* = 1	31.29 (21.55–45.61***) *p* = 2.4e−64	5.09 (3.85–6.65***) *p* = 9.5e−25
*HLA-DRB1*	NM_002124.3:c.118_122del	7	4.09	0.15	0.07	0.24	59.12 (19.62–213.94***) *p* = 1.5e−13	17.57 (7.84–38.33***) *p* = 5.7e−10	2.03 (0.04–18.14) *p* = 0.48	0.60 (0.01–3.82) *p* = 1
*HLA-DRB1*	NM_002124.3:c.126_127insTTAAGTTT	7	2.83	–	0.05	0.13	54.73 (16.83–230.03***) *p* = 1.4e−12	22.29 (9.16–55.30***) *p* = 1.9e−10	–	–
*HLA-DRB1*	NM_002124.3:c.C301T	4	0.99	0.00	0.00	0.06	Inf (12.54–Inf***) *p* = 5e−06	16.51 (3.79–72.00***) *p* = 0.00023	0.00 (0.00–Inf) *p* = 1	0.00 (0.00–14.41) *p* = 1
*NTRK1*	NM_001012331.1:c.C1792T	6	1.03	6.01	0.03	1.16	34.72 (9.25–130.03***) *p* = 1.8e−06	0.89 (0.32–1.98) *p* = 1	212.31 (91.71–602.69***) *p* = 1.3e−68	5.43 (3.94–7.38***) *p* = 1.5e−20
*NTRK1*	NM_001012331.1:c.G1820T	5	0.86	6.02	0.03	1.13	28.88 (6.95–113.95***) *p* = 2.2e−05	0.76 (0.24–1.81) *p* = 0.76	212.73 (91.75–603.88***) *p* = 1.3e−68	5.58 (4.05–7.59***) *p* = 5.2e−21
*CHIC2*	NM_012110.4:c.G36T	5	0.86	–	0.03	0.37	28.44 (6.85–112.29***) *p* = 2.2e−05	2.33 (0.73–5.74) *p* = 0.11	–	–
*CNTRL*	NM_001330762.2:c.G1009A	5	0.86	5.52	0.02	0.82	57.55 (11.17–368.15***) *p* = 3.4e−06	1.05 (0.34–2.53) *p* = 0.86	385.11 (124.23–2015.72***) *p* = 2.9e−65	7.08 (5.02–9.85***) *p* = 4.2e−23
*ISX*	NM_001303508.2:c.G248A	5	0.86	5.15	0.04	1.41	21.66 (5.56–75.41***) *p* = 4.9e−05	0.61 (0.20–1.45) *p* = 0.46	135.30 (63.17–330.64***) *p* = 1.9e−56	3.81 (2.72–5.24***) *p* = 6.8e−13
***MYO5A***	**NM_000259.3:c.A3960T**	**5**	**0.86**	**–**	**0.01**	**0.19**	84.79 (13.85–894.94***) *p* = 1.8e−06	4.64 (1.42–11.92*) *p* = 0.01	**–**	**–**
*TET2*	NM_001127208.2:c.C1088T	5	1.03	4.94	0.04	0.82	29.69 (8.22–103.70***) *p* = 2.9e−06	1.26 (0.45–2.82) *p* = 0.57	147.52 (65.91–392.69***) *p* = 1.2e−54	6.26 (4.38–8.81***) *p* = 3e−19
***BRD7***	**NM_001173984.3:c.A44C**	**4**	**0.80**	**–**	**–**	**–**	**–**	**–**	**–**	**–**
*CBFA2T3*	NM_005187.6:c.G308C	4	0.69	9.55	0.01	0.53	69.07 (9.88–783.26***) *p* = 2.5e−05	1.31 (0.35–3.49) *p* = 0.62	1036.93 (280.67–8192.00***) *p* = 6.9e−118	19.97 (14.70–27.09***) *p* = 1.8e−66
*DCC*	NM_005215.4:c.A3578G	4	0.69	–	0.10	0.65	7.08 (1.74–21.57**) *p* = 0.0065	1.06 (0.28–2.80) *p* = 0.85	–	–
*PTPRB*	NM_001206971.3:c.C3412T	4	0.69	2.15	0.01	0.44	67.61 (9.67–732.95***) *p* = 2.6e−05	1.58 (0.42–4.23) *p* = 0.42	214.17 (51.95–1908.91***) *p* = 5.6e−25	5.01 (2.90–8.29***) *p* = 6.8e−08
*RNF213*	NM_001256071.3:c.C12847A	3	0.52	6.55	0.00	0.39	Inf (14.24–Inf***) *p* = 5.3e−05	1.32 (0.27–4.04) *p* = 0.58	Inf (366.99–Inf***) *p* = 4.5e−83	17.80 (12.36–25.52***) *p* = 3.4e−44
*RNF213*	NM_001256071.3:c.C13945G	4	0.69	8.37	0.00	0.53	Inf (22.77–Inf***) *p* = 2.7e−06	1.31 (0.35–3.48) *p* = 0.62	Inf (481.56–Inf***) *p* = 2.7e−106	17.22 (12.54–23.59***) *p* = 2.5e−55
***CLIP1***	**NM_001247997.1:c.C80T**	**3**	**0.52**	**1.29**	**0.00**	**0.04**	Inf (14.10–Inf***) *p* = 5.3e−05	14.11 (2.35–62.12**) *p* = 0.0043	Inf (59.62–Inf***) *p* = 1.1e−16	35.48 (12.85–106.41***) *p* = 5.3e−12
***CUX1***	**NM_001202543.2:c.C3317T**	**3**	**0.52**	**–**	**0.01**	**0.09**	51.86 (5.92–612.07***) *p* = 0.00038	5.75 (1.08–19.95*) *p* = 0.03	**–**	**–**
*FBLN2*	NM_001998.3:c.G2569T	3	0.52	0.22	0.00	1.34	Inf (12.79–Inf***) *p* = 6.5e−05	0.38 (0.08–1.14) *p* = 0.13	Inf (3.62–Inf**) *p* = 0.0031	0.16 (0.02–0.58**) *p* = 0.0011
***GNAS***	**NM_016592.4:c.A266G**	**3**	**0.52**	**–**	**0.00**	**0.03**	Inf (13.11–Inf***) *p* = 6.2e−05	16.06 (2.59–75.51**) *p* = 0.0034	**–**	**–**
*MAF*	NM_001031804.3:c.G655T	3	4.81	–	0.00	–	Inf (13.95–Inf***) *p* = 3.4e−06	–	–	–
***MGA***	**NM_001080541.2:c.C1883A**	**3**	**0.52**	**1.54**	**0.00**	**0.08**	Inf (12.57–Inf***) *p* = 6.7e−05	6.19 (1.15–21.71*) *p* = 0.026	Inf (64.84–Inf***) *p* = 7.9e−19	18.55 (8.36–40.72***) *p* = 1.6e−11
*MLLT1*	NM_005934.4:c.G889A	3	0.52	3.00	0.03	0.20	17.85 (2.77–91.79**) *p* = 0.0029	2.54 (0.48–8.66) *p* = 0.19	106.35 (40.38–354.94***) *p* = 2.6e−31	15.11 (8.10–28.74***) *p* = 1.1e−17
***NBEA***	**NM_015678.4:c.C2317A**	**3**	**0.99**	**–**	**–**	**–**	**–**	**–**	**–**	**–**
*NUP214*	NM_001318324.2:c.A2263G	3	0.52	1.29	0.02	1.01	34.26 (4.58–255.75***) *p* = 0.00076	0.51 (0.10–1.51) *p* = 0.48	85.96 (23.16–475.65***) *p* = 4e−14	1.28 (0.65–2.29) *p* = 0.46
*PDGFRB*	NM_001355016.2:c.G1261A	3	0.52	3.54	0.00	0.18	Inf (14.24–Inf***) *p* = 5.3e−05	2.84 (0.56–9.04) *p* = 0.14	Inf (186.85–Inf***) *p* = 5.1e−45	20.03 (12.01–33.36***) *p* = 7.7e−26
*RABEP1*	NM_001291581.2:c.G1755C	3	0.52	4.51	0.00	0.36	Inf (13.90–Inf***) *p* = 5.3e−05	1.46 (0.29–4.47) *p* = 0.56	Inf (236.36–Inf***) *p* = 8.9e−57	13.22 (8.71–19.86***) *p* = 2.5e−27
*ZNF479*	NM_001370129.1:c.T1421C	3	0.52	2.58	0.00	0.54	Inf (13.94–Inf***) *p* = 5.3e−05	0.96 (0.19–2.90) *p* = 1	Inf (128.88–Inf***) *p* = 1.2e−32	4.89 (2.98–7.73***) *p* = 5.3e−09

–, indicates that a variant is not reported in a cohort, *Inf* infinity

The number of asterisks correspond to the *p* values of the Benjamini–Hochberg corrected two-tailed Fisher’s exact test: “***” if less than 0.001; “**” if less than 0.01; “*” if less than 0.05

Variants selected for Sanger validation are shown in bold font

### Case–control analysis using a breast cancer case cohort from dbGaP

Case–control analysis for the 49 germline variants identified from our Singapore breast cancer cohort was repeated using a case cohort from dbGaP (phs000822.v1.p1) against the same control cohorts (Table 3). Only 34 of our 49 variants were found in phs000822.v1.p1. Of these 34 variants, 26 were significantly enriched in phs000822.v1.p1 when compared against gnomAD (EAS) while eight did not reach statistical significance. Next, comparison of the 34 variants with SG10K_Health found 26 significantly enriched in phs000822.v1.p1, four unreported in SG10K_Health, and another four did not reach significance. These two sets of comparison were generally concordant, as 23 of the 26 significantly enriched phs000822.v1.p1 versus gnomAD (EAS) were also significantly enriched in comparison against SG10K_Health (Table 3). Altogether, 14 variants were significantly enriched in cases, or missing in the control cohorts, across all four sets of case–control comparisons. These variants were found in 89 out of 290 breast cancer patients (30.7%) where 24 of the 89 cases had more than one pathogenic variant (Additional file 4: Table S3).

### Variant validation by Sanger sequencing

**Fig. 3:**
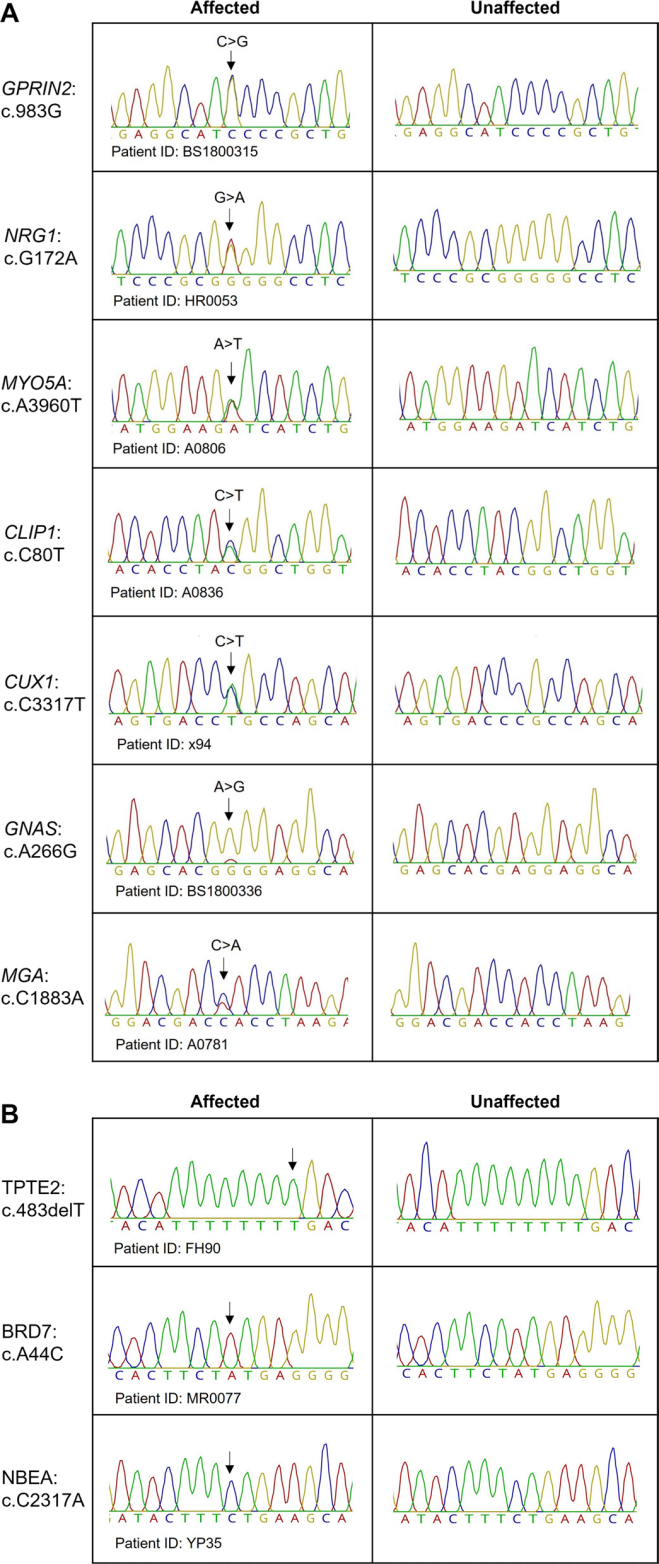
Sanger sequencing validation of variants identified by whole-exome sequencing. Representative sequencing chromatograms showing the different variants found in our breast cancer patients and of an unaffected control. **A** Seven variants were confirmed by Sanger sequencing. **B** Three variants failed to be validated by Sanger sequencing. Arrows indicate the position of the variant

Four of 14 significant variants were excluded from Sanger sequencing validation as these variants lie in highly repetitive regions (*KMT2C*, *MUC4*, and *MAF*) or highly polymorphic regions (*HLA-DRB1)*. Seven of the remaining 10 variants, including *GPRIN2* c.983G*, NRG1* c.G172A*, MYO5A* c.A3960T*, CLIP1* c.C80T, *CUX1* c.C3317T*, GNAS* c.A266G and *MGA* c.C1883A, were confirmed by Sanger sequencing. However, variants in *TPTE2*, *NBEA*, and *BRD7* could not be validated by Sanger sequencing, suggesting that these variants were likely false positives (Fig. 3).

## Discussion

Here, we report the largest WES study on germline DNA from Asian breast cancer patients who had undergone cancer risk assessment and were *BRCA1* and *BRCA2* mutation-negative. The approach that was taken was to select only pathogenic variants that showed a statistically significant difference against gnomAD East-Asian controls and Singapore controls. This was followed by an additional prioritization step of selecting only variants occurring in well documented cancer genes such as those listed in COSMIC, NCG and cancer driver gene databases [9–11].

In total, we have identified 49 rare pathogenic germline variants in 37 genes which were significantly enriched in breast cancer patients. These were all predicted to be pathogenic using in silico tools and all had a minor allele frequency of less than 1% or were unreported in gnomAD (EAS). We further validated these results with an independent United States-based case cohort obtained from dbGaP, of 466 early-onset breast cancer patients. Across four sets of comparisons involving two case and two control cohorts, 14 variants were consistently enriched in breast cancer cases (Table 3).

Of these 14 variants, seven variants in *GPRIN2*, *NRG1*, *MYO5A*, *CLIP1*, *CUX1*, *GNAS*, and *MGA* were confirmed by Sanger sequencing. To the best of our knowledge, these specific germline variants identified here have not been reported in any cancer-related studies thus far. However, their respective gene functions have been implicated in many cancer types [12–17]. The *NRG1* nonsynonymous SNV (rs113317778) lies in an immunoglobulin-like domain, while other affected residues in *GPRIN2* (rs4445576), *CUX1* (rs782176246), *GNAS* (rs563844600), and *MGA* (rs61736074) are located within a protein disordered region, where it lacks a stable tertiary structure and adopts different structural conformations [18–20]. Interestingly, a computational study has predicted the mutation in *GPRIN2* (p.S328C) to generate new microstructural elements in the disordered region and may disrupt protein functions or protein–protein interactions [20]. Other exome sequencing studies have also identified a damaging germline mutation in *GPRIN2* (p.A233S) in Iranian patients with familial esophageal squamous cell carcinoma (ESCC) [21] as well as somatic mutations in melanoma samples [22].

Additionally, a frameshift deletion variant in *TPTE2* (c.483delT) and two nonsynonymous SNVs in *NBEA* (c.C2317A) and *BRD7* (c.A44C) could not be confirmed by Sanger sequencing. *NBEA* has segmental duplications on chr15, while *BRD7* is mapped to segmentally duplicated regions on chr3 and chr6. Furthermore, the *TPTE2* variant is within a short 8-nucleotides homopolymer, and it has two segmental duplications on chrY and chr21 [23]. Due to high sequence similarities, sequenced reads which arise from segmental duplications may be wrongly aligned and result in false-positive variant calls.

Seven nonsynonymous SNVs in *RNF43*, *HLA-B*, *ERBB3*, *NTRK1*, *TET2*, and *DCC* identified here, have previously been implicated in various cancer types Additional file 4: Table S4. For example, the *HLA-B* c.A161G variant, which was detected in 9 patients (3.1%) here, was also found to be associated with high-grade cervical preinvasive lesions and invasive cervical cancer in a recent genome-wide association study [24]. A different study reported that the *ERBB3* c.A3355T variant was significantly associated with poor survival in ER-positive cases [25]. Nonetheless, none of these variants were significantly enriched in our case–control analyses.

Of our 49 variants, 4.1% (2/49) were classified as pathogenic and 61.2% (30/49) as VUS by InterVar, respectively. This high VUS rate is consistent with our previous study and that of others on Asian populations [26, 27]. In a large US study on germline genetic testing, Asian patients had approximately two-fold more VUS compared to non-Hispanic White patients, at a VUS rate above 40% [27]. These substantially higher VUS rates in Asians may reflect the underlying lack of variant data from Asian control populations available for variant reclassification.

Besides the variants identified in this current study, WES has been performed to detect candidate variants in *BRCA*-negative patients from other populations. In a study on 7 families from France, Italy, Netherlands, Australia and Spain, investigators found 12 variants in genes involved in DNA repair, cell proliferation and survival, or cell cycle regulation [28]. Sequencing of 52 individuals from 17 Greek families with HBOC and further validation in additional cohorts from Canada, TCGA and the UK Biobank, led to the prioritization of missense variants in the *SETBP1* and *c7orf34* genes [29]. In another European study, 54 *BRCA*-negative families from Belgium underwent WES and 44% harbored variants in known cancer predisposition genes. In particular, it was observed that nonsense variants in cancer-associated genes involved in DNA repair were enriched in breast cancer patients as compared to controls [30]. From 113 families from Tunisia, eight *BRCA*-negative unrelated patients were selected for WES. Of 24 genes that were prioritized from WES data, five were selected based on their significant association with survival, as determined from analysis using TCGA data [31]. Notably, the strategies for the prioritization and filtering of genes/variants differ between studies with differing variants identified. It is possible that these variants could be population-specific or low penetrance variants.

Our study has limitations. We had used an independent breast cancer cohort of US patients with early-onset breast cancer [35 years or younger] from dbGaP to validate the frequency of the 49 variants discovered in our cohort that were found to be associated with breast cancer. However, 17 of the 49 variants were not present in this dbGaP case cohort, possibly due to differences in genetic ancestry between the populations. Hence, further studies in additional Asian as well as European populations are necessary to validate the variants described in this current study. Secondly, DNA samples from family members of our cases were not available for segregation analysis. Thirdly, due to limited access to the SG10K_Health cohort, we had used the gnomAD (EAS) population for variant filtering. The gnomAD (EAS) cohort is comprised of individuals of Korean, Japanese and Chinese descent, whereas our study population were South-East Asians, mainly of Chinese, Malay and Indian ethnicity. Nonetheless, the gnomAD (EAS) was the most suitable publicly available control population available, and thus was selected.

## Conclusions

In summary, the current study has identified 49 pathogenic variants in 37 genes associated with breast cancer predisposition, many of which have not been previously documented. Our study provides new insights into the genetic susceptibility to BC, and it is imperative that further studies in additional populations of diverse ethnic background be undertaken to determine the frequency of these variants, and to confirm their association with BC risk.

## Materials and methods

### Study participants

Two hundred and ninety breast cancer patients who fulfilled one or more of the following criteria were selected for WES: 1. having a family history of breast cancer in first- and/or second-degree relatives; 2. having bilateral breast cancer; and, 3. having early-onset breast cancer at the age of 40 years or below (Additional file 1: Fig. S1) [26]. Written informed consent was obtained from all participants and the study was approved by the SingHealth Centralised Institutional Review Board (CIRB Ref: 2018/2147).

### Whole-exome sequencing

Genomic DNA was isolated from peripheral blood samples, collected from breast cancer patients as described previously [32, 33]. Samples for sequencing and libraries were prepared according to Agilent SureSelect Human All Exon V6 kit (Agilent Technologies, CA, USA) and the library preparation and enrichment were carried out according to Agilent SureSelect protocols. Enriched samples with paired-end sequencing (2X150 bp) were performed on the Illumina NovaSeq 6000 platform. Variants were aligned and called with Illumina DRAGEN version 3.5.7 on the BaseSpace Sequence Hub cloud platform [34], with median 80 × coverage per base.

### Prioritization and filtering of variants

The variants were annotated for their transcript effects, CADD v1.3 scaled score [35], and gnomAD minor allele frequencies using ANNOVAR [36]. CADD v1.3 indel scores were filled in manually using the CADD web server. The American College of Medical Genetics and the Association of Molecular Pathology (ACMG-AMP) classifications were obtained using InterVar [37]. We removed variants which did not pass DRAGEN’s default quality control checks, variants with gnomAD (EAS) MAF greater than 1%, and variants found in only two or fewer patients. Frameshift indels, stop-gains; and nonsynonymous SNVs with scaled CADD v1.3 score greater than 20 were chosen for further analysis. A CADD score of 20 and above represents the top 1% of pathogenic variants as scored by CADD.

### Prioritization of candidate genes

From the genes of our prioritized variants, we selected only known or candidate cancer genes as listed by the NCG [9]. These genes were then further curated for those that were strongly implicated in cancer in at least one other cancer gene database: the COSMIC database [10], cancer driver genes based on nucleotide context [11], and computationally discovered and experimentally validated cancer driver genes [38] (Additional file 4: Table S1).

### Manual checking with IGV

All prioritized variants were manually checked with Integrative Genomics Viewer (IGV) [39], except those in highly repetitive regions in *MUC4* or *KMT2C*, or highly polymorphic genes *HLA-A* or *HLA-DRB1,* as their alignments were too complex (Additional file 2: Fig. S2). Variants suspected to be false positives were excluded (Additional file 3: Fig. S3).

### Case–control analysis

Case–control analysis for the variants was performed for two breast cancer cohorts (cases described in this study and the phs000822.v1.p1 dataset from dbGaP) and two control cohorts (gnomAD (EAS) and SG10K_Health). The dataset from dbGaP is a breast cancer dataset of 466 patients with early-onset breast cancer (diagnosed on or before the age of 35) from the United States of America. The gnomAD (EAS) cohort (gnomAD v2.1.1) comprises 9,977 individuals of East Asian descent while the SG10K_Health cohort consists of whole genomes from 9,770 healthy Chinese, Indian, and Malay volunteers from Singapore [8].

### Polymerase chain reaction and Sanger sequencing

Variants that were significant by case–control analysis were validated by polymerase chain reaction (PCR) and Sanger sequencing. PCR primer sets were designed using Primer-BLAST [40]. DNA amplification by PCR was performed using HotStartTaq (Qiagen, Venlo, Netherlands) or Q5 High-Fidelity (New England Biolabs, Ipswich, MA, USA) DNA polymerase, as described in the manufacturer’s protocol. Primer sequences and their respective cycling conditions are listed in Additional file 4: Table S5. The PCR products were then analyzed by 2% agarose gel electrophoresis and purified with ExoSAP-IT Express (Thermo Scientific, USA) prior to sequencing. Cycle sequencing reactions were performed using BigDye Terminator v3.1 kit (Applied Biosystems, Foster City, CA) and the sequencing products were analyzed on a Genetic Analyzer. DNA sequences were visualized and aligned using Geneious Prime version 2022.1.

### Statistical analysis

For case–control analyses, a two-sided Fisher’s exact test was used and *p* values were adjusted for multiple testing using the Benjamini–Hochberg method [41].

## Supplementary Information

**Additional file 1**: **Fig.** **S1**. Detailed distribution of age at breast cancer diagnosis and family history. Patients with age of diagnosis above 40 years of age but who are without a family history of any cancer, and patients with both age of diagnosis ≤ 40 years of age and also > 40 years of age; had bilateral breast cancer.

**Additional file 2**: **Fig. S2.** Representative IGV screenshots of unambiguous versus ambiguous alignments. **A** A heterozygous SNV with equal support for both reference and alternate bases; **B** deletion, as indicated by clear gaps in the read alignment; and **C** insertion, as represented by a thin vertical line flanked by mapped bases on both sides. Red boxes indicate where the variants are expected to appear. In comparison, the heterozygous nonsynonymous SNVs **D** MUC4 NM_018406.7:c.G8461A **E** KMT2C NM_170606.3:c.C2689T **F** KMT2C NM_170606.3:c.C2710T and **G** HLA-DRB1 NM_002124.3:c.C301T have fewer reads supporting the alternate base; the frameshift deletions **H** HLA-A NM_001242758.1:c.268delA and **I** HLA-DRB1 NM_002124.3:c.118_122del are not associated with any obvious gaps in read alignments; nor is the frameshift insertion **J** HLA-DRB1 NM_002124.3:c.126_127insTTAAGTTT represented by insertions in its read alignments.

**Additional file 3**: **Fig. S3. **Representative IGV screenshots of alignments supporting two likely–false positive frameshift insertions. Panel **A** shows the alignment for PABPC1 NM_002568.4:c.1336_1337insACCTCATC and **B** for CIC NM_015125.4:c.4778_4779insGG. Red boxes indicate where the insertion would have been expected to appear, red arrows point to the soft-clipped alignments which support the existence these frameshift insertions. **C** Reads supporting the PABPC1 insertion map partially to both PABPC1 and PABPC3 (reverse complement) genes on reference genome loci NC_000008.10:101,719,206-101,719,234 and NC_000013.11:25,097,536-25,097,508, respectively.

**Additional file 4**: **Supplementary Table 1.** Total number of potentially pathogenic variants discovered in each prioritized gene; and support for these genes across different cancer gene databases. **Supplementary Table 2.** Patient IDs for the patients with rare pathogenic variants in each gene. **Supplementary Table 3.** Clinical features and pathogenic variants identified in 89 breast cancer patients. **Supplementary Table 4.** Involvement in cancer for seven of our selected variants, as reported in the literature. **Supplementary Table 5.** PCR primers and cycling conditions used for Sanger sequencing.

## Data Availability

The datasets used and analyzed during the current study are available from the corresponding author on reasonable request.

## Abbreviations

ACMG-AMP: American College of Medical Genetics and Association for Molecular Pathology
BC: Breast cancer
CADD: Combined Annotation-Dependent Depletion
COSMIC: Catalogue of Somatic Mutations in Cancer
dbGaP: Database of Genotypes and Phenotypes
DRAGEN: Dynamic Read Analysis for GENomics
EAS: East-Asian
ESCC: Esophageal squamous cell carcinoma
FDR: False discovery rate
gnomAD: Genome Aggregation Database
IGV: Integrative Genomics Viewer
MAF: Minor allele frequency
NCG: Network of Cancer Genes
PCR: Polymerase chain reaction
SNV: Single nucleotide variant
VUS: Variants of uncertain significance
WES: Whole-exome sequencing

## References

[1] Sung H, Ferlay J, Siegel RL, Laversanne M, Soerjomataram I, Jemal A (2021). Global cancer statistics 2020: GLOBOCAN estimates of incidence and mortality worldwide for 36 cancers in 185 countries. CA Cancer J Clin.

[2] Rahman N, Stratton MR (1998). The genetics of breast cancer susceptibility. Annu Rev Genet.

[3] Daly MB, Pilarski R, Yurgelun MB, Berry MP, Buys SS, Dickson P (2020). NCCN guidelines insights: genetic/familial high-risk assessment: breast, ovarian, and pancreatic, Version 1.2020. J Natl Compr Cancer Netw.

[4] Melchor L, Benítez J (2013). The complex genetic landscape of familial breast cancer. Hum Genet.

[5] Nielsen FC, van Overeem HT, Sørensen CS (2016). Hereditary breast and ovarian cancer: new genes in confined pathways. Nat Rev Cancer.

[6] Kiiski JI, Pelttari LM, Khan S, Freysteinsdottir ES, Reynisdottir I, Hart SN (2014). Exome sequencing identifies FANCM as a susceptibility gene for triple-negative breast cancer. Proc Natl Acad Sci.

[7] Chandler MR, Bilgili EP, Merner ND (2016). A review of whole-exome sequencing efforts toward hereditary breast cancer susceptibility gene discovery. Hum Mutat.

[8] Precision Health Research, Singapore SG10K. Precision Health Research, Singapore (PRECISE) [Internet]. [cited 2021 Jun 24]. Available from: https://web.archive.org/web/20210624043126/https://www.npm.sg/collaborate/partners/sg10k/

[9] Repana D, Nulsen J, Dressler L, Bortolomeazzi M, Venkata SK, Tourna A (2019). The Network of Cancer Genes (NCG): a comprehensive catalogue of known and candidate cancer genes from cancer sequencing screens. Genome Biol.

[10] Bamford S, Dawson E, Forbes S, Clements J, Pettett R, Dogan A (2004). The COSMIC (Catalogue of Somatic Mutations in Cancer) database and website. Br J Cancer.

[11] Dietlein F, Weghorn D, Taylor-Weiner A, Richters A, Reardon B, Liu D (2020). Identification of cancer driver genes based on nucleotide context. Nat Genet.

[12] Jones MR, Williamson LM, Topham JT, Lee MKC, Goytain A, Ho J (2019). NRG1 gene fusions are recurrent, clinically actionable gene rearrangements in KRAS wild-type pancreatic ductal adenocarcinoma. Clin Cancer Res.

[13] Sato N, Fujishima F, Nakamura Y, Aoyama Y, Onodera Y, Ozawa Y (2018). Myosin 5a regulates tumor migration and epithelial-mesenchymal transition in esophageal squamous cell carcinoma: utility as a prognostic factor. Hum Pathol.

[14] Izumi H, Matsumoto S, Liu J, Tanaka K, Mori S, Hayashi K (2021). The CLIP1-LTK fusion is an oncogenic driver in non-small-cell lung cancer. Nature.

[15] Ramdzan ZM, Vickridge E, Faraco CCF, Nepveu A (2021). CUT domain proteins in DNA repair and cancer. Cancers (Basel).

[16] Jin X, Zhu L, Cui Z, Tang J, Xie M, Ren G (2019). Elevated expression of GNAS promotes breast cancer cell proliferation and migration via the PI3K/AKT/Snail1/E-cadherin axis. Clin Transl Oncol.

[17] Cancer Genome Atlas Research Network (2014). Comprehensive molecular profiling of lung adenocarcinoma. Nature.

[18] UniProt Consortium (2021). UniProt: the universal protein knowledgebase in 2021. Nucleic Acids Res.

[19] van der Lee R, Buljan M, Lang B, Weatheritt RJ, Daughdrill GW, Dunker AK (2014). Classification of intrinsically disordered regions and proteins. Chem Rev.

[20] Li C, Clark LVT, Zhang R, Porebski BT, McCoey JM, Borg NA (2018). Structural capacitance in protein evolution and human diseases. J Mol Biol.

[21] Khalilipour N, Baranova A, Jebelli A, Heravi-Moussavi A, Bruskin S, Abbaszadegan MR (2018). Familial esophageal squamous cell carcinoma with damaging rare/germline mutations in KCNJ12/KCNJ18 and GPRIN2 genes. Cancer Genet.

[22] Wei X, Walia V, Lin JC, Teer JK, Prickett TD, Gartner J (2011). Exome sequencing identifies GRIN2A as frequently mutated in melanoma. Nat Genet.

[23] Kent WJ, Sugnet CW, Furey TS, Roskin KM, Pringle TH, Zahler AM (2002). The human genome browser at UCSC. Genome Res.

[24] Bowden SJ, Bodinier B, Kalliala I, Zuber V, Vuckovic D, Doulgeraki T (2021). Genetic variation in cervical preinvasive and invasive disease: a genome-wide association study. Lancet Oncol.

[25] Varadi V, Bevier M, Grzybowska E, Johansson R, Enquist-Olsson K, Henriksson R (2012). Genetic variation in ALCAM and other chromosomal instability genes in breast cancer survival. Breast Cancer Res Treat.

[26] Wong ESY, Shekar S, Met-Domestici M, Chan C, Sze M, Yap YS (2016). Inherited breast cancer predisposition in Asians: multigene panel testing outcomes from Singapore. NPJ Genomic Med.

[27] Kurian AW, Ward KC, Abrahamse P, Bondarenko I, Hamilton AS, Deapen D (2021). Time trends in receipt of germline genetic testing and results for women diagnosed with breast cancer or ovarian cancer, 2012–2019. J Clin Oncol.

[28] Gracia-Aznarez FJ, Fernandez V, Pita G, Peterlongo P, Dominguez O, de la Hoya M (2013). Whole exome sequencing suggests much of non-BRCA1/BRCA2 familial breast cancer is due to moderate and low penetrance susceptibility alleles. PLoS One..

[29] Glentis S, Dimopoulos AC, Rouskas K, Ntritsos G, Evangelou E, Narod SA (2019). Exome sequencing in BRCA1- and BRCA2-negative greek families identifies MDM1 and NBEAL1 as candidate risk genes for hereditary breast cancer. Front Genet.

[30] Shahi RB, De Brakeleer S, Caljon B, Pauwels I, Bonduelle M, Joris S (2019). Identification of candidate cancer predisposing variants by performing whole-exome sequencing on index patients from BRCA1 and BRCA2-negative breast cancer families. BMC Cancer.

[31] BenAyed-Guerfali D, Kifagi C, BenKridis-Rejeb W, Ammous-Boukhris N, Ayedi W, Khanfir A (2022). The identification by exome sequencing of candidate genes in BRCA-negative tunisian patients at a high risk of hereditary breast/ovarian cancer. Genes (Basel).

[32] Ang P, Lim IHK, Lee T-C, Luo J-T, Ong DCT, Tan PH (2007). BRCA1 and BRCA2 mutations in an Asian clinic-based population detected using a comprehensive strategy. Cancer Epidemiol Biomarkers Prev.

[33] Chan M, Ji SM, Yeo ZX, Gan L, Yap E, Yap YS (2012). Development of a next-generation sequencing method for BRCA mutation screening. J Mol Diagnostics.

[34] Miller NA, Farrow EG, Gibson M, Willig LK, Twist G, Yoo B (2015). A 26-hour system of highly sensitive whole genome sequencing for emergency management of genetic diseases. Genome Med.

[35] Rentzsch P, Schubach M, Shendure J, Kircher M (2021). CADD-Splice—improving genome-wide variant effect prediction using deep learning-derived splice scores. Genome Med.

[36] Wang K, Li M, Hakonarson H (2010). ANNOVAR: functional annotation of genetic variants from high-throughput sequencing data. Nucleic Acids Res.

[37] Li Q, Wang K (2017). InterVar: clinical interpretation of genetic variants by the 2015 ACMG-AMP guidelines. Am J Hum Genet.

[38] Bailey MH, Tokheim C, Porta-Pardo E, Sengupta S, Bertrand D, Weerasinghe A (2018). Comprehensive characterization of cancer driver genes and mutations. Cell.

[39] Robinson JT, Thorvaldsdóttir H, Wenger AM, Zehir A, Mesirov JP (2017). Variant review with the integrative genomics viewer. Cancer Res.

[40] Ye J, Coulouris G, Zaretskaya I, Cutcutache I, Rozen S, Madden TL (2012). Primer-BLAST: a tool to design target-specific primers for polymerase chain reaction. BMC Bioinform.

[41] Benjamini Y, Hochberg Y (1995). Controlling the false discovery rate: a practical and powerful approach to multiple testing. J R Stat Soc Ser B.

