# Correction: Whole-exome sequencing of *BRCA*-negative breast cancer patients and case–control analyses identify variants associated with breast cancer susceptibility

**DOI:** 10.1186/s40246-022-00443-7

**Published:** 2022-12-19

**Authors:** Ning Yuan Lee, Melissa Hum, Aseervatham Anusha Amali, Wei Kiat Lim, Matthew Wong, Matthew Khine Myint, Ru Jin Tay, Pei-Yi Ong, Jens Samol, Chia Wei Lim, Peter Ang, Min-Han Tan, Soo-Chin Lee, Ann S. G. Lee

**Affiliations:** 1 Division of Cellular and Molecular Research, Humphrey Oei Institute of Cancer Research, National Cancer Centre Singapore, 11 Hospital Crescent, Singapore, 169610 Singapore; 2 Lucence Diagnostics Pte Ltd, 211 Henderson Road, Singapore, 159552 Singapore; 3 Department of Hematology-Oncology, National University Cancer Institute, Singapore (NCIS), National University Health System, 5 Lower Kent Ridge Road, Singapore, 119074 Singapore; 4 Medical Oncology Department, Tan Tock Seng Hospital, 11 Jalan Tan Tock Seng, Singapore, 308433 Singapore; 5 Johns Hopkins University, Baltimore, MD 21218 USA; 6 Department of Personalised Medicine, Tan Tock Seng Hospital, 11 Jalan Tan Tock Seng, Singapore, 308433 Singapore; 7 Oncocare Cancer Centre, Gleneagles Medical Centre, 6 Napier Road, Singapore, 258499 Singapore; 8 Department of Medicine, Yong Loo Lin School of Medicine, National University of Singapore, 10 Medical Dr, Singapore, 117597 Singapore; 9 Cancer Science Institute, Singapore (CSI), National University of Singapore, 14 Medical Dr, Singapore, 117599 Singapore; 10 Department of Physiology, Yong Loo Lin School of Medicine, National University of Singapore, 2 Medical Drive, Singapore, 117593 Singapore; 11 SingHealth Duke-NUS Oncology Academic Clinical Programme (ONCO ACP), Duke-NUS Graduate Medical School, 8 College Road, Singapore, 169857 Singapore

**Correction: Human Genomics (2022) 16:61** https://doi.org/10.1186/s40246-022-00435-7

Following publication of the original article [1], the authors reported Fig. 1 is not the updated version. The correct Fig. 1 has been provided in this Correction.

**Fig. 1:**
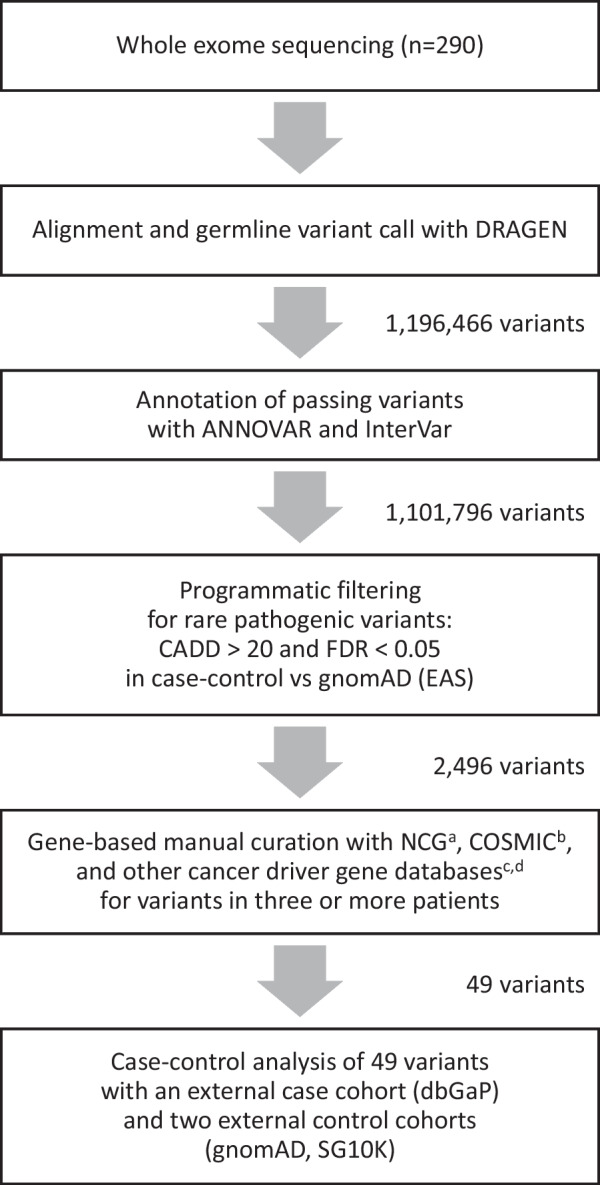
Study design for the selection of variants and genes. ^a^List of known or candidate cancer genes in the Network of Cancer Genes [9]. ^b^The Cancer Gene Census list of the Catalogue of Somatic Mutations in Cancer (COSMIC) [10]. ^c^List of cancer driver genes from Bailey et al. [38]. ^d^List of cancer driver genes inferred with nucleotide context from Dietlein et al. [11]

The original article [1] has been corrected.
