# Supplementary figures and images for: Whole-exome sequencing of BRCA-negative breast cancer patients and case–control analyses identify variants associated with breast cancer susceptibility
Source: Hum Genomics. 2022 Nov 23;16:61. doi: 10.1186/s40246-022-00435-7 (PMC9685974; doi:10.1186/s40246-022-00435-7)

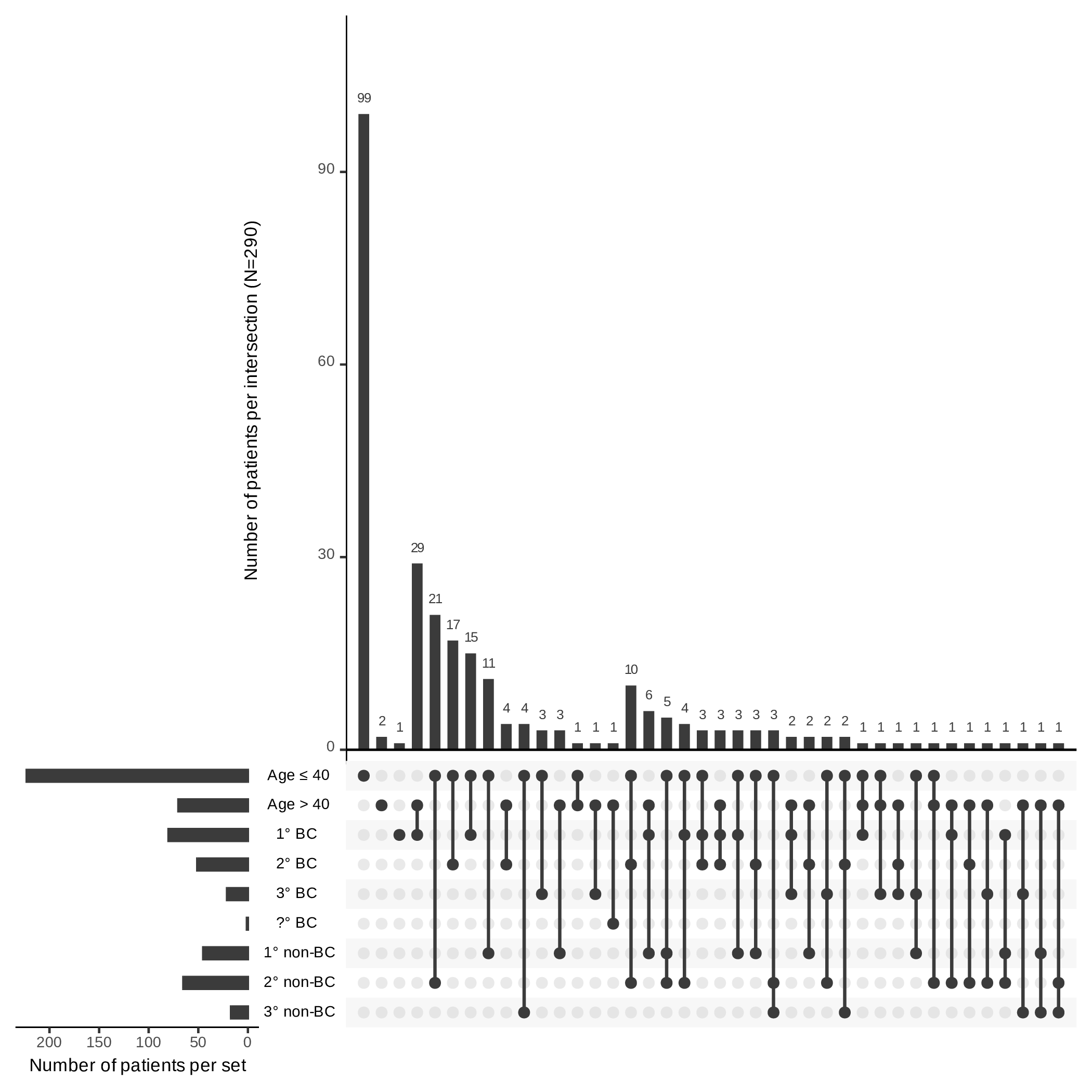

Supplement: Supplementary file 1 — Additional file 1: Fig. S1. Detailed distribution of age at breast cancer diagnosis and family history. Patients with age of diagnosis above 40 years of age but who are without a family history of any cancer, and patients with both age of diagnosis ≤ 40 years of age and also > 40 years of age; had bilateral breast cancer. [file 40246_2022_435_MOESM1_ESM.png]

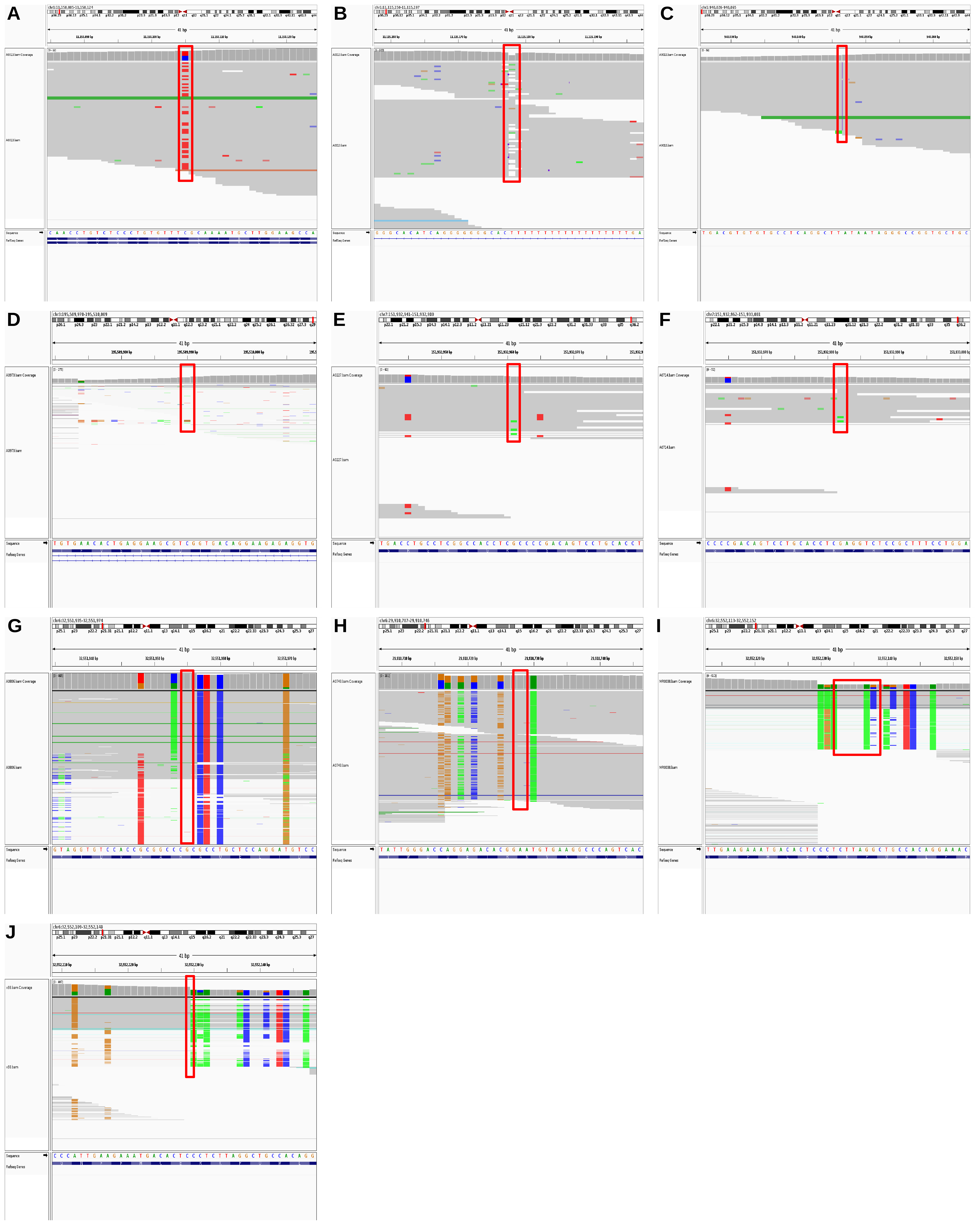

Supplement: Supplementary file 2 — Additional file 2: Fig. S2. Representative IGV screenshots of unambiguous versus ambiguous alignments. A A heterozygous SNV with equal support for both reference and alternate bases; B deletion, as indicated by clear gaps in the read alignment; and C insertion, as represented by a thin vertical line flanked by mapped bases on both sides. Red boxes indicate where the variants are expected to appear. In comparison, the heterozygous nonsynonymous SNVs D MUC4 NM_018406.7:c.G8461A E KMT2C NM_170606.3:c.C2689T F KMT2C NM_170606.3:c.C2710T and G HLA-DRB1 NM_002124.3:c.C301T have fewer reads supporting the alternate base; the frameshift deletions H HLA-A NM_001242758.1:c.268delA and I HLA-DRB1 NM_002124.3:c.118_122del are not associated with any obvious gaps in read alignments; nor is the frameshift insertion J HLA-DRB1 NM_002124.3:c.126_127insTTAAGTTT represented by insertions in its read alignments. [file 40246_2022_435_MOESM2_ESM.png]

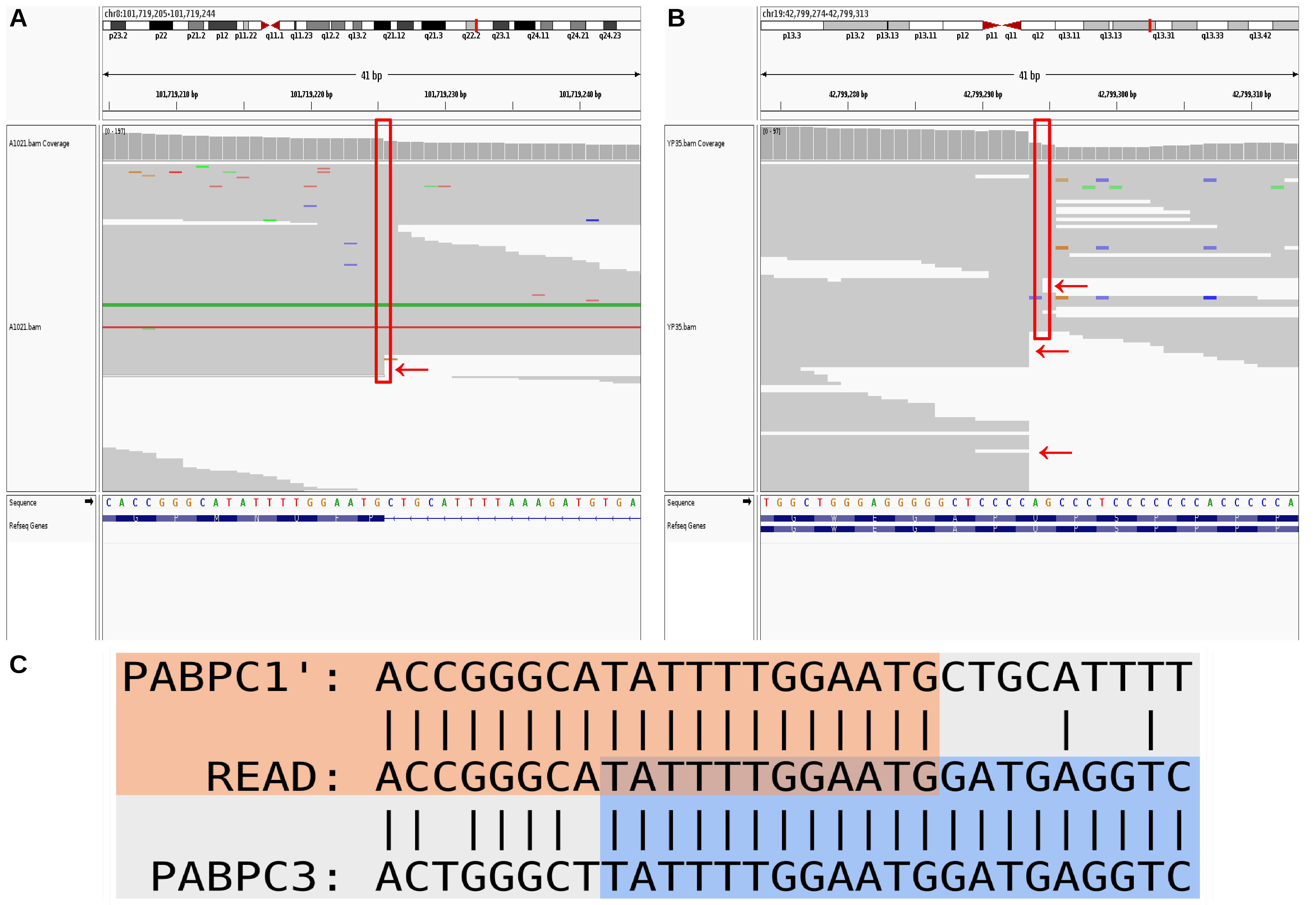

Supplement: Supplementary file 3 — Additional file 3: Fig. S3. Representative IGV screenshots of alignments supporting two likely–false positive frameshift insertions. Panel A shows the alignment for PABPC1 NM_002568.4:c.1336_1337insACCTCATC and B for CIC NM_015125.4:c.4778_4779insGG. Red boxes indicate where the insertion would have been expected to appear, red arrows point to the soft-clipped alignments which support the existence these frameshift insertions. C Reads supporting the PABPC1 insertion map partially to both PABPC1 and PABPC3 (reverse complement) genes on reference genome loci NC_000008.10:101,719,206-101,719,234 and NC_000013.11:25,097,536-25,097,508, respectively. [file 40246_2022_435_MOESM3_ESM.png]
