# Supplementary material for: Whole-exome sequencing of BRCA-negative breast cancer patients and case–control analyses identify variants associated with breast cancer susceptibility
Source: Hum Genomics. 2022 Nov 23;16:61. doi: 10.1186/s40246-022-00435-7 (PMC9685974; doi:10.1186/s40246-022-00435-7)
Supplement: Supplementary file 4 — Additional file 4: Supplementary Table 1. Total number of potentially pathogenic variants discovered in each prioritized gene; and support for these genes across different cancer gene databases. Supplementary Table 2. Patient IDs for the patients with rare pathogenic variants in each gene. Supplementary Table 3. Clinical features and pathogenic variants identified in 89 breast cancer patients. Supplementary Table 4. Involvement in cancer for seven of our selected variants, as reported in the literature. Supplementary Table 5. PCR primers and cycling conditions used for Sanger sequencing. [file 40246_2022_435_MOESM4_ESM.docx]

**Supplementary Table 1. Total number of potentially pathogenic variants discovered in each prioritized gene; and support for these genes across different cancer gene databases.**

| **Hugo**  **Symbol** | **FR DEL** | **FR INS** | **NSV** | **SG** | **Total** | **Mutated**  **Samples** | **% of Cohort with Mutation** | **COSMIC**  **Somatic^a^** | **NCG known^b^** | **NCG candidate list**  **(n = 104)^c^** | **NCG candidate list**  **(n = 711)^d^** | **Driver Genes^e^** | **Literature**  **Support^f^** |
| --- | --- | --- | --- | --- | --- | --- | --- | --- | --- | --- | --- | --- | --- |
| *KMT2C* | 0 | 0 | 0 | 35 | 35 | 33 | 11.38 | Y | Y |  |  | Y | level A |
| *MUC4* | 0 | 0 | 16 | 0 | 16 | 16 | 5.52 | Y | Y |  |  |  |  |
| *RNF43* | 0 | 0 | 21 | 0 | 21 | 20 | 6.9 | Y | Y |  |  | Y | level A |
| *GPRIN2* | 0 | 0 | 17 | 0 | 17 | 17 | 5.86 |  |  |  | Y |  | level B |
| *H3F3A* | 0 | 0 | 14 | 0 | 14 | 14 | 4.83 | Y | Y |  |  | Y |  |
| *AKAP9* | 0 | 0 | 11 | 0 | 11 | 11 | 3.79 | Y | Y |  |  |  | level A |
| *ROS1* | 0 | 0 | 8 | 0 | 8 | 8 | 2.76 | Y | Y |  |  |  |  |
| *TPTE2* | 10 | 0 | 0 | 0 | 10 | 10 | 3.45 |  |  | Y | Y |  | level B |
| *NRG1* | 0 | 0 | 9 | 0 | 9 | 9 | 3.1 | Y | Y |  |  |  |  |
| *HLA-DRB1* | 7 | 7 | 4 | 0 | 18 | 7 | 2.41 |  |  |  | Y |  | level B |
| *HLA-B* | 0 | 0 | 9 | 0 | 9 | 9 | 3.1 |  |  | Y | Y | Y | level B |
| *ELN* | 0 | 0 | 8 | 0 | 8 | 8 | 2.76 | Y | Y |  |  |  |  |
| *HLA-A* | 8 | 0 | 27 | 8 | 43 | 8 | 2.76 | Y | Y |  |  | Y | level A |
| *TET2* | 0 | 0 | 5 | 0 | 5 | 5 | 1.72 | Y | Y |  |  | Y | level A |
| *NTRK1* | 0 | 0 | 11 | 0 | 11 | 6 | 2.07 | Y | Y |  |  |  |  |
| *DCC* | 0 | 0 | 4 | 0 | 4 | 4 | 1.38 | Y | Y |  |  |  |  |
| *CHIC2* | 0 | 0 | 5 | 0 | 5 | 5 | 1.72 | Y | Y |  |  |  |  |
| *CNTRL* | 0 | 0 | 5 | 0 | 5 | 5 | 1.72 | Y | Y |  |  |  |  |
| *ISX* | 0 | 0 | 5 | 0 | 5 | 5 | 1.72 | Y | Y |  |  |  |  |
| *MYO5A* | 0 | 0 | 5 | 0 | 5 | 5 | 1.72 | Y | Y |  |  |  |  |
| *NBEA* | 0 | 0 | 3 | 0 | 3 | 3 | 1.03 | Y | Y |  |  |  | level A |
| *RNF213* | 0 | 0 | 7 | 0 | 7 | 4 | 1.38 | Y | Y |  |  |  |  |
| *CBFA2T3* | 0 | 0 | 4 | 0 | 4 | 4 | 1.38 | Y | Y |  |  |  |  |
| *PTPRB* | 0 | 0 | 4 | 0 | 4 | 4 | 1.38 | Y | Y |  |  |  |  |
| *CLIP1* | 0 | 0 | 3 | 0 | 3 | 3 | 1.03 | Y | Y |  |  |  | level A |
| *BRD7* | 0 | 0 | 4 | 0 | 4 | 4 | 1.38 |  |  | Y | Y | Y | level B |
| *FBLN2* | 0 | 0 | 3 | 0 | 3 | 3 | 1.03 | Y | Y |  |  |  |  |
| *MAF* | 0 | 0 | 3 | 0 | 3 | 3 | 1.03 | Y | Y |  |  |  |  |
| *MLLT1* | 0 | 0 | 3 | 0 | 3 | 3 | 1.03 | Y | Y |  |  |  |  |
| *NUP214* | 0 | 0 | 3 | 0 | 3 | 3 | 1.03 | Y | Y |  |  |  |  |
| *PDGFRB* | 0 | 0 | 3 | 0 | 3 | 3 | 1.03 | Y | Y |  |  |  |  |
| *RABEP1* | 0 | 0 | 3 | 0 | 3 | 3 | 1.03 | Y | Y |  |  |  |  |
| *CUX1* | 0 | 0 | 3 | 0 | 3 | 3 | 1.03 | Y | Y |  |  |  | level A |
| *ERBB3* | 0 | 0 | 8 | 0 | 8 | 8 | 2.76 | Y | Y |  |  | Y | level A |
| *GNAS* | 0 | 0 | 3 | 0 | 3 | 3 | 1.03 | Y | Y |  |  | Y | level A |
| *ZNF479* | 0 | 0 | 3 | 0 | 3 | 3 | 1.03 | Y | Y |  |  |  | level A |
| *MGA* | 0 | 0 | 3 | 0 | 3 | 3 | 1.03 |  |  | Y | Y | Y | level B |

**Abbreviations:** FR DEL = frameshift deletions, FR INS = frameshift insertions, NSV = nonsynonymous variants, SG = stopgains.

^a^ “Y” indicates that the gene contains known somatic mutations causally implicated in cancer, according to the COSMIC v94 Cancer Gene Census (10).

^b^ “Y” indicates that the gene is a known cancer gene according to the Network of Cancer Genes v6.0 (9).

^c^ “Y” indicates that the gene is listed (n = 104) as a candidate cancer gene with strong support according to the Network of Cancer Genes v6.0 (9).

^d^ “Y” indicates that the gene is listed (n = 711) as a candidate cancer gene with strong support according to the Network of Cancer Genes v6.0 (9).

^e^ “Y” indicates that the gene appears in the curated list of cancer driver genes (38).

^f^ “Y” indicates that the gene appears in the list of cancer driver genes derived using nucleotide context (11). Evidence levels range from A (strongest) to D (weakest).

**Supplementary Table 2.** **Patient IDs for the patients with rare pathogenic variants in each gene.**

| **Gene** | **Patient ID** |
| --- | --- |
| *KMT2C* | A0227, A0587, A0623, A0661, A0714, A0785, A0792, A0827, A0853, A0881, A0909, A0951, A0999, BS1800682, BS1800740, BS1800793, FH81, FH90, FH91, FH92, HCR003, MR0077a, SG1800868, SG1801020, SG1801271, x206, x235, x2365633, x66, x69, x83, YP24, YP9 |
| *RNF43* | A0669, A0717, A0728, A0836, A0909, BS1800315, HCR003, LR0037, SG1800961, SG1801020, SG1801192, SG1801236, SG1900119, x154, x171, x181, x205, x207, x208, x93 |
| *GPRIN2* | A0668, A0728, A0781, A0806, A0909, A0989, A1021, BS1800315, HCR003, HR0053, SG1801023, SG1801184, SG1900119, x207, x208, x2365633, x237 |
| *MUC4* | A0973, A1007, BS1800315, BS1800682, BS1800740, BS1800793, FH96, MR0031, MR0038, SG1800109, SG1900119, x135, x81, YP23, YP42, YP68 |
| *H3F3A* | A0989, BLSG1800992, BLSG1801173, HR0044, MR0077a, SG1800109, x161, x93, YP18, YP2, YP25, YP42, YP52, YP66 |
| *AKAP9* | A0665, A0890, A1005, BS1800450, BS1800571, FH95, x171, x241, YP13, YP68, YP7 |
| *TPTE2* | A0427, A0614, A0674, A0792, A0951, A1021, FH90, x118, x156, YP39 |
| *HLA-B* | A0698, A0728, A0758, A0804, A0945, BS1800508, SG1900119, YP55, YP68 |
| *NRG1* | A0227, A0608, A0820, A0953, FH97, HR0053, SG1800961, SG1801365, x231 |
| *ELN* | A0669, A0674, A0684, A0781, A0957, A0963, SG1800961, x181 |
| *ERBB3* | A0728, A0806, BS1800336, HCR010, SG1800961, x161, x93, YP18 |
| *HLA-A* | A0743, BLSG1801173, BS1800616, LR0037, MR0019, SG1801192, x161, x222 |
| *ROS1* | A0669, A0781, A0820, A1045, BS1800315, BS1800336, HCR010, LR0037 |
| *HLA-DRB1* | A0657, A0806, BLSG1800992, MR0038, x174, x93, x980131 |
| *NTRK1* | A0645, A0717, A1007, SG1801023, x231, x94 |
| *CHIC2* | LR0075, SG1800109, x148, x82, x980131 |
| *CNTRL* | A0717, A0770, A0836, HR0053, x208 |
| *ISX* | A0909, A0989, SG1801023, SG1801236, x231 |
| *MYO5A* | A0806, BS1800315, BS1800336, HCR003, HCR010 |
| *TET2* | A0909, HCR003, HR0053, x237, x93 |
| *BRD7* | A0877, A1056, MR0077, YP23 |
| *CBFA2T3* | A0989, BS1800336, HCR010, SG1800961 |
| *DCC* | A0830, x171, x231, YP2 |
| *PTPRB* | A0836, HR0053, x181, x208 |
| *RNF213* | SG1801023, SG1801236, x154, x205 |
| *CLIP1* | A0836, SG1801236, SG1900119 |
| *CUX1* | A0963, BLSG1801173, x94 |
| *FBLN2* | A0674, A0890, SG1801020 |
| *GNAS* | BS1800336, HCR010, SG1801020 |
| *MAF* | A0811, BS1800571, YP53 |
| *MGA* | A0781, x94, YP68 |
| *MLLT1* | BS1800398, HCR003, x181 |
| *NBEA* | MR0003, MR0031, YP35 |
| *NUP214* | A0909, SG1800961, x154 |
| *PDGFRB* | A0909, BS1800336, HCR010 |
| *RABEP1* | HCR003, HR0053, SG1801236 |
| *ZNF479* | A0674, A0811, SG1801236 |

**Supplementary Table 3.** **Clinical features and pathogenic variants identified in 89 breast cancer patients**

| **Gene** | **Variant** | **ID** | **Ethnicity** | **Family History (Age at diagnosis)^b^** | **Age at diagnosis (years)** | **Histology** | **ER/PR/Her2 status** |
| --- | --- | --- | --- | --- | --- | --- | --- |
| *KMT2C* | c.2710C>T | A0227^a^ | M | Mat Un Col Ca (50s); Mat great GM Br Ca (60s) | 32 | Mucinous | ER+/PR+/Her2- |
|  |  | A0587 | C | Pat Au Pr Ca (60s); Pat GF Col Ca (78) | 36 | IDC | ER+/PR+/Her2- |
|  |  | A0623 | O | No FH Ca | 27 | Unk | ER+/PR+/Her2- |
|  |  | A0661 | O | Mat Co leukemia (40s); Pat Un NPC (50s) | 35 | ILC | ER+/PR+/Her2- |
|  |  | A0792^a^ | C | Mo Ov and Col Ca (51); Pat Au Col Ca (60s) | 33 | IDC | ER-/PR-/Her2+ |
|  |  | A0827 | C | Pat Au Ut Ca | 36 | IDC | ER+/PR+/Her2- |
|  |  | A0853 | C | Mo Br Ca (50s); Mat GM Br Ca (40s) | 39 | IDC | ER-/PR-/Her2+ |
|  |  | A0881 | C | Pat Un Pa Ca (53); Pat GF Lung Ca (53); Mat Co Col Ca (32) | 39 | Unk | ER Unk/PR Unk/Her2 Unk |
|  |  | A0951^a^ | C | Pat great GM Ga Ca (60s) | 40 | IDC | ER+/PR+/Her2- |
|  |  | A0999 | M | Mat Au Cer Ca (40s); Mo’s Mat Co Br Ca (38) | 37 | Mucinous | ER+/PR+/Her2- |
|  |  | BS1800682^a^ | C | Fa Col Ca; Sis Br Ca; Co Br Ca | 52 | Unk | ER-/PR-/Her2- |
|  |  | BS1800793^a^ | C | Mat GM Br Ca (28) | 28 | Unk | ER+/PR Unk/ Her2 Unk |
|  |  | FH81 | C | Sis Br Ca | 60 | IDC | ER-/PR-/Her2+ |
|  |  | FH90^a^ | C | Co Br Ca; Co Br Ca | 47 | IDC | ER+/PR-/Her2- |
|  |  | FH91 | C | Sis Ca Unk | 45 | IDC | ER+/PR+/Her2- |
|  |  | FH92 | C | Mat Au Other Ca; Pat Au Other Ca | 42 | IDC, DCIS | ER+/PR+/Her2- |
|  |  | HCR003^a^ | Cau | Mo Endo Ca | 44 | DCIS | ER+/PR-/Her2 Unk |
|  |  | MR0077a | C | Sis Br Ca (45); Pat Au Bil Br Ca (48,56) | 39 | IDC | ER+/PR-/Her2- |
|  |  | SG1800868 | I | Dau Br Ca | 56 | Unk | ER-/PR-/Her2+ |
|  |  | SG1801271 | C | Mo Br Ca; Mat GM Lung Ca; Bro lymphoma | 56 | Unk | ER Unk/PR Unk/Her2 Unk |
|  |  | x206 | C | Mo Col and Br Ca (50); Mat Un Neck Ca (50); Pat Au Ga Ca (50) | 47 | DCIS | ER+/PR+/Her2 Unk |
|  |  | x235 | F | Pat Au Br Ca (37); Mat GF’s Sis Br (40) and Ov Ca (60) | 25 | Unk | ER Unk/PR Unk/Her2 Unk |
|  |  | x2365633^a^ | O | No FH Ca | 29 | IDC | ER Unk/PR Unk/Her2 Unk |
|  |  | x66 | C | Dau Ov Ca (25) | 55 | IDC | ER+/PR+/Her2+ |
|  |  | x69 | C | Sis Br Ca (46); Pat Au Br Ca (60); Pat Au Br Ca (65) | 46 | IDC | ER+/PR+/Her2+ |
|  |  | x83 | C | No FH Ca | 19 | IDC | ER+/PR+/Her2- |
|  |  | YP24 | C | No FH Ca | 35 | IDC | ER+/PR+/Her2- |
|  |  | YP9 | C | Fa Ca Unk (70) | 40 | IDC | ER+/PR-/Her2+ |
| *GPRIN2* | c.983C>G | A0668 | I | No FH Ca | 29 | IDC | ER-/PR+/Her2- |
|  |  | A0728 | O | Fa lymphoma (62); Pat GF Pros Ca (80s) | 28 | IDC | ER+/PR+/Her2- |
|  |  | A0781^a^ | I | No FH Ca | 38 | IDC | ER-/PR+/Her2+ |
|  |  | A0806^a^ | O | Pat Co Br Ca (65); Mat Co Br Ca (75) | 24 | IDC | ER+/PR+/Her2- |
|  |  | A0909 | O | No FH Ca | 37 | IDC | ER+/PR+/Her2+ |
|  |  | A0989 | O | No FH Ca | 35 | IDC | ER+/PR+/Her2- |
|  |  | A1021^a^ | I | Pat Au Br Ca (50s) | 29 | IDC | ER+/PR+/Her2- |
|  |  | BS1800315^a^ | Cau | Mat Au Br Ca (65); Mat Au Br Ca (30); Fa Pa Ca (67) | 36 | Unk | ER+/PR+/Her2+ |
|  |  | HCR003^a^ | Cau | Mo Endo Ca | 44 | DCIS | ER+/PR-/Her2 Unk |
|  |  | HR0053^a^ | O | Mo Bil Br Ca (42) and Ov Ca (52); Mat GM Br Ca (35) | 32 | IDC | ER-/PR-/Her2- |
|  |  | SG1801023 | Cau | Mo Br Ca | 38 | IDC | ER-/PR+/Her2- |
|  |  | SG1801184 | M | Mat GM Br Ca; Mat Au Gy Ca; Mo Col Ca | 40 | Unk | ER Unk/PR Unk/Her2 Unk |
|  |  | SG1900119^a^ | Cau | Sis Br Ca | 57 | IDC | ER+/PR+/Her2 Unk |
|  |  | x207 | I | Unk FH | 30 | IDC, DCIS | ER+/PR+/Her2+ |
|  |  | x208 | I | Sis Br Ca (40) | 56 | Unk | ER Unk/PR Unk/Her2 Unk |
|  |  | x2365633^a^ | O | No FH Ca | 29 | IDC | ER Unk/PR Unk/Her2 Unk |
|  |  | x237 | Cau | Pat Au Br Ca (46); Pat Au Br Ca (48); Pat great GM Br Ca; Mat GM Col Ca (83) | 33 | IDC, DCIS | ER+/PR-/Her2- |
| *MUC4* | c.8461G>A | A0973 | C | Fa Col (60) and Li Ca (65); Pat Un Lung Ca (70s) | 38 | IDC | ER-/PR-/Her2+ |
|  |  | A1007 | O | Fa RC Ca (60s); Pat Au Ute Ca (55); Pat Au Skin Ca (44); Pat Co Col Ca (44); Mat GF leukemia; Mat Un Col Ca (60); Mat Co Thy Ca (30s) | 40 | Multifocal IDC | ER+/PR+/Her2- |
|  |  | BS1800315^a^ | Cau | Mat Au Br Ca (65); Mat Au Br Ca (30); Fa Pa Ca (67) | 36 | Unk | ER+/PR+/Her2+ |
|  |  | BS1800682^a^ | C | Fa Col Ca; Sis Br Ca; Co Br Ca | 52 | Unk | ER-/PR-/Her2- |
|  |  | BS1800740 | C | Sis Br Ca (50); Sis Br Ca (50); Mat Co Br Ca (50) | 57 | DCIS | ER+/PR+/Her2 Equiv |
|  |  | BS1800793^a^ | C | Mat GM Br Ca (28) | 28 | Unk | ER+/PR Unk/ Her2 Unk |
|  |  | FH96 | C | Sis Ca Unk (45); Co Ca Unk (45) | 45 | IDC | ER+/PR+/Her2- |
|  |  | MR0031^a^ | C | Sis Br Ca (37) | 53 | IDC | ER+/PR+/Her2+ |
|  |  | MR0038^a^ | C | No FH Ca | 28 | IDC | ER+/PR+/Her2+ |
|  |  | SG1800109 | F | Sis MM; Sis Br Ca; Bro Pros Ca; Bro Col Ca; Fa Col Ca | 75 | Unk | ER Unk/PR Unk/Her2 Unk |
|  |  | SG1900119^a^ | Cau | Sis Br Ca | 57 | IDC | ER+/PR+/Her2 Unk |
|  |  | x135 | C | Sis Br Ca (50) | 45 | DCIS, IDC | ER Unk/PR Unk/Her2 Unk |
|  |  | x81 | C | No FH Ca | 30 | IDC | ER-/PR-/Her2+ |
|  |  | YP23^a^ | C | No FH Ca | 40 | No case note | ER+/PR+/Her2- |
|  |  | YP42 | C | No FH Ca | 28 | IDC | ER+/PR-/Her2- |
|  |  | YP68^a^ | M | No FH Ca | 40 | TLC | ER+/PR+/Her2- |
| *TPTE2* | c.483delT | A0427 | C | Mo Br Ca (34,54) | 34 | IDC | ER+/PR+/Her2- |
|  |  | A0614 | C | No FH Ca | 35 | IDC | ER+/PR+/Her2 Unk |
|  |  | A0674 | I | No FH Ca | 40 | IDC | ER+/PR+/Her2- |
|  |  | A0792^a^ | C | Mo Ov and Col Ca (51); Pat Au Col Ca (60s) | 33 | IDC | ER-/PR-/Her2+ |
|  |  | A0951^a^ | C | Pat great GM Ga Ca (60s) | 40 | ILC | ER+/PR+/Her2- |
|  |  | A1021^a^ | I | Pat Au Br Ca (50s) | 29 | IDC | ER+/PR+/Her2- |
|  |  | FH90^a^ | C | Co Br Ca; Co Br Ca | 47 | IDC | ER+/PR-/Her2- |
|  |  | x118 | C | No FH Ca | 33 | MBC | ER-/PR+/Her2- |
|  |  | x156 | C | No FH Ca | 35 | Unk | ER Unk/PR Unk/Her2 Unk |
|  |  | YP39 | C | No FH Ca | 33 | IDC | ER+/PR+/Her2- |
| *NRG1* | c.172G>A | A0227^a^ | M | Mat Un Col Ca (50s); Mat great GM Br Ca (60s) | 32 | Mucinous | ER+/PR+/Her2- |
|  |  | A0608 | O | Mat GF laryngeal Ca (83) | 34 | IDC | ER+/PR+/Her2+ |
|  |  | A0820 | I | Mo’s Pat Co Li Ca (50s); Mo’s Pat Co Brain tumor (60s); Mo’s Pat Co Spinal Ca (60s); Mo’s Pat Co Col Ca (58); Mo’s Pat Co Li Ca (30s); Mo’s Pat Co SG Ca (30s); Mo’s Pat Co Throat Ca (64); Mo’s Pat Co Br Ca (59); Son of Mo’s Pat Co Col Ca (55) | 36 | IDC | ER+/PR+/Her2+ |
|  |  | A0953 | C | No FH Ca | 29 | IDC | ER+/PR+/Her2- |
|  |  | FH97 | M | Sis Br Ca (50); Sis Ft Ca | 52 | DCIS | ER-/PR-/Her2 Unk |
|  |  | HR0053^a^ | O | Mo Bil Br Ca (42) Ov Ca (52); Mat GM Br Ca (35) | 32 | IDC | ER-/PR-/Her2- |
|  |  | SG1800961 | G | Pat GM Br Ca | 45 | Unk | ER+/PR+/Her2+ |
|  |  | SG1801365 | B | Mat Co Br Ca; Mat Au Ga Ca | 50 | Unk | ER Unk/PR Unk/Her2 Unk |
|  |  | x231 | I | Mat Un Lung Ca (60); Mat Au Br Ca (60); Pat GF Lung Ca (80) | 36 | IDC, DCIS | ER+/PR+/Her2+ |
| *HLA-DRB1* | c.126_127insTTAAGTTT | A0657 | C | No FH Ca | 29 | IDC | ER-/PR-/Her2+ |
|  |  | A0806^a^ | O | Pat Co Br Ca (65); Mat Co Br Ca (75) | 24 | IDC | ER+/PR+/Her2- |
|  |  | BLSG1800992 | V | No FH Ca | 34 | IDC | ER+/PR+/Her2- |
|  |  | MR0038^a^ | C | No FH Ca | 28 | IDC | ER+/PR+/Her2+ |
|  |  | x174 | C | No FH Ca | 36 | IDC | ER+/PR-/Her2+ |
|  |  | x93 | I | No FH Ca | 34 | IDC | ER-/PR-/Her2- |
|  |  | x980131 | C | No FH Ca | 28 | IDC, DCIS | ER-/PR-/Her2 Unk |
| *MYO5A* | c.3960A>T | A0806^a^ | O | Pat Co Br Ca (65); Mat Co Br Ca (75) | 24 | IDC | ER+/PR+/Her2- |
|  |  | BS1800315^a^ | Cau | Mat Au Br Ca (65); Mat Au Br Ca (30); Fa Pa Ca (67) | 36 | Unk | ER+/PR+/Her2+ |
|  |  | BS1800336^a^ | A | Mat Au Br Ca (70); Sis Br Ca (55) | 58 | Unk | ER+/PR+/Her2 Equiv |
|  |  | HCR003^a^ | Cau | Mo Endo Ca | 44 | DCIS | ER+/PR-/Her2 Unk |
|  |  | HCR010^a^ | A | Sis Br Ca | 57 | ILC | ER+/PR+/Her2- |
| *BRD7* | c.44A>C | A0877 | C | Mat Un Li Ca (60s); Mat Un Li Ca and NPC (60s); Mat GF Li Ca (60s) | 32 | IDC | ER-/PR+/Her2- |
|  |  | A1056 | C | No FH Ca | 38 | IDC | ER+/PR+/Her2- |
|  |  | MR0077 | C | Sis Br Ca (39); Pat Au Bil Br Ca (48,56) | 45 | IDC | ER+/PR+/Her2- |
|  |  | YP23^a^ | C | No FH Ca | 40 | No case note | ER+/PR+/Her2- |
| *CLIP1* | c.80C>T | A0836 | I | Mat GM Br Ca (75); Mat great GM Li Ca (50s) | 28 | IDC | ER+/PR-/Her2- |
|  |  | SG1801236 | Unk | Mat GM Br Ca; Pat GM Ov Ca | 40 | Unk | ER+/PR+/Her2- |
|  |  | SG1900119^a^ | Cau | Sis Br Ca | 57 | IDC | ER+/PR+/Her2 Unk |
| *CUX1* | c.3317C>T | A0963 | M | Mat Au Blood Ca (40s) | 28 | Mucinous | ER+/PR+/Her2- |
|  |  | BLSG1801173 | F | No FH Ca | 33 | IDC | ER+/PR+/Her2- |
|  |  | x94^a^ | C | No FH Ca | 30 | IDC | ER+/PR+/Her2- |
| *GNAS* | c.266A>G | BS1800336^a^ | A | Mat Au Br Ca (70); Sis Br Ca (55) | 58 | Unk | ER+/PR+/Her2 Equiv |
|  |  | HCR010^a^ | A | Sis Br Ca | 57 | ILC | ER+/PR+/Her2- |
|  |  | SG1801020 | T | Mo Br Ca; Sis Br Ca | 51 | Unk | ER Unk/PR Unk/Her2 Unk |
| *MAF* | c.655G>T | A0811 | O | No FH Ca | 38 | IDC | ER-/PR-/Her2- |
|  |  | BS1800571 | C | Mo Col and Br Ca (48); Mat GF Col Ca | 40 | Unk | ER-/PR-/Her2- |
|  |  | YP53 | C | No FH Ca | 39 | IDC | ER-/PR-/Her2+ |
| *MGA* | c.1883C>A | A0781^a^ | I | No FH Ca | 38 | IDC | ER-/PR+/Her2+ |
|  |  | x94^a^ | C | No FH Ca | 30 | IDC | ER+/PR+/Her2- |
|  |  | YP68^a^ | M | No FH Ca | 40 | TLC | ER+/PR+/Her2- |
| *NBEA* | c.2317C>A | MR0003 | C | Sis Br Ca (46); Mo Br Ca (55) | 45 | IDC | ER+/PR+/Her2- |
|  |  | MR0031^a^ | C | Sis Br Ca (37) | 53 | IDC | ER+/PR+/Her2+ |
|  |  | YP35 | C | No FH Ca | 37 | ILC | ER+/PR-/Her2- |

**Abbreviations:**

A, Arab; Au, aunt; B, Bengali; Bil, bilateral; Br, breast; C, Chinese; Ca, cancer; Cau, Caucasian; Cer, cervical; Co, cousin; Col, colorectal; Dau, daughter; DCIS, ductal carcinoma in-situ; Endo, endometrial; Equiv, equivocal; ER, oestrogen receptor; F, Filipino; Fa, father; FH, family history; Ft, fallopian tube; G, Gujarati; Ga, gastric; GF, grandfather; GM, grandmother; Gy, gynecologic; I, Indonesian; IDC, invasive ductal carcinoma; ILC, invasive lobular carcinoma; Ki, kidney; Li, liver; Mat, maternal; MBC, medullary breast carcinoma; MM, multiple myeloma; Mo, mother; NPC, nasopharyngeal cancer; O, others; Ov, ovarian; Pa, pancreatic; Pat, paternal; PR, progesterone receptor; Pros, prostate; RC, renal cell; SG, salivary gland; Sis, sister; T, Tamil; Thy, thyroid; TLC, tubulolobular carcinoma; Un, uncle; Unk, unknown; Ute, uterine; V, Vietnamese.

^a^ Patients with more than one pathogenic variant.

^b^ Age at diagnosis of family member is provided if available.

**Supplementary Table 4.** **Involvement in cancer for seven of our selected variants, as reported in the literature.**

| **Gene** | **HGVS** | **RefSNP** | **Disease** | **Reference** |
| --- | --- | --- | --- | --- |
| *RNF43* | c.G647A | rs34523089 | Gastric cancer | Cell Mol Gastroenterol Hepatol. 2021, 11(4):1071-94 |
| *HLA-B* | c.A161G | rs9266183 | Cervical intraepithelial neoplasia grade 3 and invasive cervical cancer | Lancet Oncol. 2021, 22(4):548-57 |
| *ERBB3* | c.A3355T | rs773123 | HER2-positive and ER-positive breast cancer | PLoS One. 2018, 13(8):e0200996; Breast Cancer Res Treat. 2012, 131(1):311-9 |
| *NTRK1* | c.C1792T | rs6336 | Sporadic medullary thyroid carcinoma, neuroblastoma | J Clin Endocrinol Metab. 1999, 84(8):2784-7; BMC Cancer. 2009, 9(1):436 |
| *NTRK1* | c.G1820T | rs6339 | Sporadic medullary thyroid carcinoma | J Clin Endocrinol Metab. 1999, 84(8):2784-7 |
| *TET2* | c.C1088T | rs17253672 | Acute myeloid leukemia, acute lymphoblastic leukemia | Medicine (Baltimore). 2020, 99(14):e19730; PeerJ. 2021, 9:e10678; J Cancer Res Clin Oncol. 2016, 142(7):1641-50 |
| *DCC* | c.A3578G | rs375401214 | Colorectal cancer | Genes, Chromosom Cancer. 2021, 60(2):61-72 |

**Supplementary Table 5. PCR primers and cycling conditions used for Sanger sequencing.**

| **Gene** | **Primer Sequence (5’ – 3’)** | **Product (bp)** | **PCR conditions** | **Enzyme** |
| --- | --- | --- | --- | --- |
| GPRIN2 | F: ACTCTGTGGCATGAGGGAGG  R: TCAGCATCCCATCGCACATC | 459 | 95°C for 15 m;  (94°C for 30 s, 63°C for 30 s, 72°C for 60 s) x 30 cycles;  72°C for 10 m | HotStarTaq DNA Polymerase (QIAGEN) |
| TPTE2 | F: GCGTGTATATTCTTCACTGGTCC  R: TGGGGATGAGTCAGTAGTTTGTG | 417 | 95°C for 15 m;  (94°C for 30 s, 61°C for 30 s, 72°C for 60 s) x 25 cycles;  72°C for 10 m | HotStarTaq DNA Polymerase (QIAGEN) |
|  | NF: GTGGGCATTTACCCAAACATTTCA  NR: ACAGGAGACAGCAGTATTTTTCTG | 274 | 95°C for 15 m;  (94°C for 30 s, 64°C for 30 s, 72°C for 60 s) x 25 cycles;  72°C for 10 m | HotStarTaq DNA Polymerase (QIAGEN) with Q-solution |
| NRG1 | F: GAGGGGAAGGAAAAGGGAGG  R: TGCACCTTTCCCTCGATCAC | 345 | 98°C for 30 s;  (98°C for 10 s, 64.6°C for 30 s, 72°C for 25 s) x 30 cycles;  72°C for 2 m | Q5 High-Fidelity DNA Polymerase (NEB) with GC Enhancer |
| MYO5A | F: ACCATCGTCACCTCATCAGC  R: CAGGGGTGAAAGTCAGGAGG | 230 | 95°C for 15 m;  (94°C for 30 s, 55°C for 30 s, 72°C for 60 s) x 30 cycles;  72°C for 10 m | HotStarTaq DNA Polymerase (QIAGEN) |
| BRD7 | F: GCGCGGGGGGCGGGCACC  R: GGAGTCGTGCCCCGAGCTGCCCGT | 171 | 95°C for 15 m;  (94°C for 30 s, 72°C for 60 s) x 30 cycles;  72°C for 10 m | HotStarTaq DNA Polymerase (QIAGEN) |
| CLIP1 | F: ATGTGAGGAGTGGGTCATGG  R: ATGCGCCTATATTCCCAGCT | 686 | 95°C for 15 m;  (94°C for 30 s, 59°C for 30 s, 72°C for 60 s) x 30 cycles;  72°C for 10 m | HotStarTaq DNA Polymerase (QIAGEN) |
| CUX1 | F: CCCCGATGAGTTCCAGTGAG  R: TTATGCCGTAGGTGTCCAGC | 219 | 95°C for 15 m;  (94°C for 30 s, 60°C for 30 s, 72°C for 60 s) x 30 cycles;  72°C for 10 m | HotStarTaq DNA Polymerase (QIAGEN) |
| GNAS | F: TCTGGCTCTCCTGCTCCAT  R: GATCGTCTTCAGGCTCGGTC | 336 | 95°C for 15 m;  (94°C for 30 s, 64.5°C for 30 s, 72°C for 60 s) x 30 cycles;  72°C for 10 m | HotStarTaq DNA Polymerase (QIAGEN) |
| MGA | F: AGGACTTGGGCAGAAAGAGA  R: CCCAGGGCTTACAGGTGTAT | 200 | 95°C for 15 m;  (94°C for 30 s, 58°C for 30 s, 72°C for 60 s) x 30 cycles;  72°C for 10 m | HotStarTaq DNA Polymerase (QIAGEN) |
| NBEA | F: GGGTGATCTACAAATTATTGGCTTC  R: ACCACAGAAAGCTATTATTTTCCGC | 225 | 95°C for 15 m;  (94°C for 30 s, 55°C for 30 s, 72°C for 60 s) x 30 cycles;  72°C for 10 m | HotStarTaq DNA Polymerase (QIAGEN) |

**Abbreviations:** F: forward; R: reverse; NF: nested forward; NR: nested reverse.
